# Graduate labour market outcomes and satisfaction with university education in Spain

**DOI:** 10.1371/journal.pone.0270643

**Published:** 2022-07-06

**Authors:** Iñaki Iriondo

**Affiliations:** Departamento de Economía Aplicada, Estructura e Historia, Facultad de Ciencias Económicas y Empresariales, Universidad Complutense de Madrid, Pozuelo de Alarcón, Madrid, Spain; University of Naples L’Orientale, ITALY

## Abstract

The objective of this paper is to analyze the association between the job placement results of graduates and the satisfaction that, retrospectively, they express about their university education. Despite the fact that, in Spain, 47.0% of the population aged 25 to 34 has a higher education degree, we have little knowledge of the determinants of university student satisfaction. In addition, most studies have focused on evaluating the university experience (teaching activity, study plans, counselling for students, or facilities) and very few of them have taken into account the influence of graduates’ labour market outcomes on their satisfaction. This issue is of particular interest in the Spanish case, considering the dysfunctional nature of the youth labor market, which has high rates of overqualification, excessive job turnover and high unemployment. The sources of statistical information used in this work include the first two editions of the "University Graduate Job Placement Survey", carried out by the Spanish National Institute of Statistics (INE) in 2014 and 2019. The methodology used in the empirical work is the propensity score matching estimator. The results of empirical analysis indicate that graduates’ job placement difficulties have a significant association with their dissatisfaction with their university experience. In particular, educational mismatch (horizontal and vertical) and, to a lesser extent, unemployment and low wages significantly increase the probability of graduates stating that, if they had to start over, they would not return to university or they would not study the same degree.

## Introduction

The objective of this article is to analyze the association between graduates’ job placement outcomes and satisfaction with their university experience in Spain. Over the last decades numerous works have been published investigating the determinants of students’ satisfaction. The impulse of this literature has been motivated, among other reasons, by massification [1], by internationalization [2] and by the greater effort that students and their families make in financing of higher education [3]. The deterioration of the youth labor market experienced in some European countries since the outbreak of the Great Recession has also revived interest in investigating the perception that graduates have about their university experience.

The introduction of tuition fees in the UK in 1998 has encouraged a change in the attitude of students, who consider the university should provide “value for money”. Initially, the substantial rise in university fees in 2012 did not have an impact on overall student satisfaction [1]. However, the replacement of maintenance grants with loans has stimulated “consumerist” attitudes towards higher education, and a growing proportion of young people study at university for career reasons [3, 4]. In addition, the increasing cost of higher education to students affect university departments, which are more involved in improving the employability of their graduates. This concern is accentuated in some fields of knowledge such as health sciences, where the cost of educational programs has increased substantially in recent decades [5, 6].

A part of the literature that analyzes the satisfaction of university students has been developed in the field of marketing [7]. From that perspective, it is assumed that consumers purchase products and services based on expectations about their value and the level of satisfaction they get from it. Universities operate in an increasingly competitive environment, which is why they are increasingly concerned about the satisfaction of their clients, the students. By analyzing the strengths and weaknesses of the service that higher education institutions provide, the student satisfaction can be improved. For instance, perceived value is the most important factor for students to be satisfied with their experience in Danish universities [7]. The perceived value of the educational service results from the comparison of what is received (the ability to manage their future career) instead of what is given (economic and personal effort in learning). Transferring the argument to the field of Economics, the satisfaction of university graduates is conditioned by the comparison between the costs and benefits of investing in human capital [8].

The measurement of the satisfaction of university students should combine the analysis of processes (teaching, study offer, student care, facilities and, among others, cultural, social or associative life) and results (job placement and professional career) [9]. Surveys of students during their studies tend to focus on processes. On the other hand, job placement surveys carried out a few years after graduation provide valuable information about results.

There is a large body of literature dedicated to analyzing the satisfaction of university students based on information related to training processes. Most studies have focused on researching the academic determinants of student satisfaction [1, 10–14]. Other articles have been devoted to measuring the satisfaction of students with specific aspects of the university experience, such as teaching methods [15], time spent studying and acquired competencies [16]. In other cases, an attempt has been made to identify patterns in student satisfaction based on whether they were enrolled part- or full-time [17] or their field of study [18]. Unfortunately, the literature that studies the association between job placement results and satisfaction with university studies is not as abundant. In addition, the association between labour market outcomes and satisfaction is not usually analyzed directly, but rather indirectly with variables that measure the preparation for a professional career [19] or the student employability [10].

One of the few papers that have looked into the problem is Whelan and McGuinness [14] who study the determinants of university student satisfaction in a sample of graduates from the European Union and Turkey. Among the explanatory variables, the effect on satisfaction of three vectors is analyzed: the student’s human capital (knowledge, skills and competences), course composition and teaching techniques and, finally, the job placement results of graduates. The authors find that wages and horizontal matching are positively associated with satisfaction, while overqualification and past periods of unemployment show a negative relationship.

Of the studies that include information about the Spanish case, mention should be first made of García-Montalvo [20], who assesses the satisfaction of university graduates from 12 countries who participated in the *Career after Higher Education project*: *a European Research Stud*y (CHEERS) by combining information from the job and educational experience of the interviewees. One of the conclusions drawn by the author is that “Spanish graduates, and particularly Italian graduates, believe their studies contributed very little to them finding a satisfactory job” (p. 227). On the other hand, the author emphasizes that Spain tops the ranking of researched countries when it comes to the likelihood of graduates choosing not to pursue higher education again if they had to make the decision at the time of completing the survey (p. 231). This result coincides with that obtained in ANECA [21], where 17% of the surveyed graduates indicated that it was “very or quite likely” that they would not have pursued higher education if they had to start over.

García-Aracil [11] once again uses CHEERS study data in order to research the variables that influence student satisfaction, such as academic advising, program content, teaching materials, grading system, elective modules on offer, equipment and, among other aspects, the use of qualifications in the workplace. The results obtained indicate that graduates who use the knowledge and skills acquired during their studies at work are more satisfied than those who do not. That is, overqualified graduates, as well as those who work outside their field of study, are less satisfied with their education.

Luque and Doña [22] find that the preparation of students for their careers and the subsequent experience of graduates in the job market have a significant association with the probability of them repeating the same studies and choosing the same university if they were starting over, the two variables they use to assess the satisfaction of graduates with their university experience. In some later works, the authors find that the variables that determine graduates’ satisfaction to a greater extent are the quality of education received and entry in the employment market, measured by remuneration, type of contract and education-job matching [23, 24]. The satisfaction of university students crucially depends on receiving a quality education that provides a credential valued in the labor market [4]. In southern European countries this statement has a special meaning, given the difficulties that young people have in finding a good job [18, 25].

In Spain, the costs of higher education have not changed substantially in recent decades and, as in other continental European countries, tuition fees are relatively low. However, the difficulties lie in the access of young graduates to qualified employment. Since the beginning of the Great Recession, the youth labor market in southern European countries has experienced a serious deterioration that has resulted in high levels of unemployment, temporary jobs and overqualification. The Spanish case is paradigmatic for having a youth unemployment rate that in 2014 reached 53.2% of persons aged 15 to 24, when the average of the European Union (EU) was 23.4% (see Table 1). In that same year, the proportion of young people with a temporary contract was 68.5% in Spain, well above the 50.2% average for the EU. Finally, overqualification measured by the percentage of people aged 20 to 64 with tertiary education who perform low-skill occupations stands at 36.4% in Spain, again well above the 21.7% average for the EU [26]. Fortunately, youth unemployment experienced a 20 percentage points drop from 2014 to 2019, although the levels of temporary employment and overqualification hardly changed [27]. For all of the above, we consider that the study of the Spanish case is of special interest given the dysfunctional nature of its youth labor market.

**Table 1:** The European labor market during the Great Recession.

	Unemployment (%)		Temporary emp. (%)		Overqualification	
	(15 to 24 years)		(15 to 24 years)		(20 to 64 years)	
TIME	2014	2019	2014	2019	2014	2019
European Union (27)	23.4	15.0	50.2	49.6	21.7	21.9
Belgium	23.2	14.2	32.2	48.5	19.9	20.0
Denmark	14.2	10.1	20.6	32.9	13.5	13.5
Germany	7.7	5.8	52.9	49.6	18.9	18.2
Ireland	23.4	12.5	34.0	34.8	29.8	30.0
Greece	52.4	35.2	30.5	30.0	26.7	32.3
Spain	53.2	32.5	68.5	68.5	36.4	36.6
France	24.2	19.5	55.3	56.8	22.2	21.7
Italy	42.7	29.2	55.6	63.4	18.7	20.2
Luxembourg	22.6	17.0	41.8	36.5	4.5	4.8
Netherlands	12.7	6.7	55.4	54.2	15.7	16.3
Austria	10.3	8.5	33.8	31.8	28.8	28.8
Portugal	34.8	18.3	60.5	62.4	12.9	14.8
Finland	20.5	17.2	49.7	46.0	18.8	17.7
Sweden	22.9	20.1	56.4	53.9	16.3	15.3
Norway	7.9	10.0	22.9	25.7	15.3	16.2

Source: EU Labour Force Survey and Experimental statistics on skill mismatch (Eurostat)

The analysis of young people’s transition into the labor market and, in particular, the problem of educational mismatch is relevant in Spain, given how difficult it is for young people to develop a satisfactory professional career [28–32]. Since Freeman’s seminal work on the phenomenon of overqualification [33], an extensive literature has been developed, dedicated, among other topics, to elaborating explanatory theories about educational mismatch [34, 35]; to measuring the effect of overqualification on wages [36–38]; to studying the temporary or permanent character of overqualification [39, 40]; and, finally, to investigating the determinants of horizontal mismatch [41]. Given the magnitude of the overqualification problem in the Spanish labor market, it was considered appropriate to include vertical and horizontal educational mismatch variables within the battery of indicators used to study the relationship between graduate job placement process results and graduates’ satisfaction with their university experience, taking the previous evidence in the literature on the subject as a reference [11, 14].

The main contribution of this article is to provide empirical evidence of the relationship of six different indicators that measure the job placement results (inactivity, unemployment, vertical and horizontal mismatch, low wages and temporary employment) with the satisfaction of university graduates, which is something that has been scarcely investigated in the literature. For this purpose, the global satisfaction of students will be analyzed based on the information provided by the 2014 and 2019 University Graduate Job Placement Survey (INE, Spanish National Institute of Statistics), one of the most complete databases available to investigate this field in Spain. We will work with variables that measure the satisfaction of graduates with their university experience four to five years after finishing their studies, that is, once they have had time to contrast the value of their education against the labor market.

From the empirical work, it is concluded that the employment outcomes of graduates have a strong association with their dissatisfaction with university education. In particular, having a job that either requires a lower level of education or is outside the field of study of the degree and, to a lesser extent, the lack of employment and earning low wages are the four variables that have a statistically significant relationship with the dissatisfaction of university graduates.

The rest of the work is structured as follows: In the next section, the data and the methodology are presented. The results of the estimates are shown in the following section, and the main conclusions are presented in the last section.

## Methods

### Description of the data

The source of statistical information used in this work includes the first two editions of the University Graduate Job Placement Survey (*Encuesta de Inserción Laboral de Titulados Universitarios*, EILU) [42] conducted by the Spanish National Institute of Statistics (INE). The EILU is a nationwide survey assessing the transition from university into the labor market of 30,379 graduates with bachelor’s degrees or first- (3 years) or second-cycle (5 years) diplomas in 2014, and 31,651 bachelor’s degree graduates and 11,483 master’s degree graduates in 2019. Data from the EILU originate in a survey that is combined with information from five administrative sources: the Municipal Register, the State Public Employment Service, the National Database of Persons with Disabilities, the Social Security and the Integrated University Information System (SIIU). The analyzed sample refers to the 2009–2010 academic year graduate cohort in the first edition, and that of bachelor’s and master’s degree graduates from the 2013–2014 academic year in the second edition.

The EILU provides information on graduates’ personal characteristics (among others, sex, age, nationality, disability, region where the university is located), their learning process (branch of knowledge, field of study and degree, university type, scholarships, satisfaction, postgraduate training), and their labor market insertion (labor force status, professional situation, occupation, the matching between education and employment, and wages [2019] or the social security wage base [2014]).

The variable of interest in this article measures graduate satisfaction with university education and it originates in the following two survey questions: 1) "*If I had to start over*, *would I go back to university*?", and 2) "*If I had to start over*, *would I study the same degree again*?*"*. Based on these two questions, a dummy variable is constructed in order to measure graduate dissatisfaction with university education. It takes a value of 1 if the graduate states that they would not go back to university or that they would not study the same degree again, and of 0 if the graduate states that, if they had to start over, they would go back to university and study the same degree again.

As noted above, the objective of this article is to analyze the satisfaction of university graduates once they have faced the job market and can check the gap between their expectations and their real experience in the world of work. In order to characterize the graduates’ employment outcomes, the following five variables will be used:

- Inactivity: it is constructed from the variable “labor force status” and it takes value 1 if the graduates are “inactive” and 0 if they are “active” (employed or unemployed). In principle, the analysis of inactivity allows us to evaluate wheher dissatisfaction with university education increases among the graduates who are less attached to the labor market. However, this variable should be handled with caution, since it includes a heterogeneous set of situations, such as “continuing studies or preparing for competitive examinations”, “retired”, “incapacity for work”, “dedicated to housework” and “other situations”.
- Unemployment: it is constructed from the variable “labor force status” and takes value 1 if the graduates are “unemployed” and 0 if they are “employed”. Through this variable, we will try to measure the relationship of dissatisfaction with being unemployed, compared to the alternative, having a job. Therefore, only graduates who are part of the active population, that is, those who wish to work, regardless of whether they have a job, are considered. The sample size is reduced as inactive graduates are excluded.
- Vertical mismatch: it measures the subjective perception of overqualification in current employment and it is constructed from the item "Most appropriate level of education for current job". The variable takes a value of 1 if the graduate states "Higher-level vocational training", "High school or intermediate vocational training" or lower-level education, such as “Lower level of vocational training”, “Compulsory secondary education” or “Primary education”, as the most appropriate level of education for the job. On the other hand, the variable takes a value of 0 if the graduate chooses "University degree (except PhD)" or "PhD" as the most appropriate level of education. The sample size is reduced since the question about educational mismatch is addressed at employed graduates.
- Horizontal mismatch: it measures the subjective perception of horizontal mismatch, that is, the situation in which the graduate performs a job outside their degree’s area of ​​knowledge. The variable is constructed from the question relating to the "Most appropriate field of study for current job" and takes a value of 1 if the graduate is working outside their field of study (in a "totally different field" or in "no particular field”) and takes the value 0 if they work in their field of study (“Exclusively in their own field of study” or in “Their own field of study or a related one”). The sample size is the same as in the vertical mismatch analysis (employed graduates).
- Low wage: data regarding graduates’ income is not comparable between waves, since the 2014 survey does not contain information on wage distribution. To address the study of wages in the first wave, we used the Social Security (SS) wage base quintiles for salaried workers from March 2014. The variable “low wage” takes a value of 1 if the graduate’s SS wage base in 2014 was in the lowest quintile, and of 0 if it was between the second and fifth quintiles. To get an idea of the income differences between quintiles, it should be taken into account that the median of the SS wage base of the first quintile was 498.8 euros per month, while that of the second quintile was 1,061.0 euros, and the median of the fifth quintile was 2,864.0 euros. It should be noted that, in 2014, the sample size for the "low wage" variable was notably reduced as a result of the lack of information on unemployed and self-employed graduates. Meanwhile, in the 2019 edition, a variable was included in order to measure “current net monthly wage” in 7 categories. Since the 2019 survey does not provide information on individual wages, the established definition of low-paid jobs as those leading to earnings below two-thirds of median earnings cannot be applied in our case [43]. The two categories with the lowest salaries (“under 700 euros” and “between 700 and 999 euros”) account for 15.8% of employed graduates, while the proportion reaches 46.8% of the total when the third category is added (“between 1,000 and 1,499 euros”). Given that the incidence of low pay in Spain is estimated at between 18.4 and 22.8% of workers [44], we have applied an ad-hoc criterion to establish that low-paid graduates are those receiving a net monthly wage of less than 1,000 euros.
- Temporary contract: it provides information on the type of contract of currently employed graduates. It takes value 1 if the graduate is in temporary employment and 0 if they are in any of the remaining categories (trainee, permanent employment, employer, self-employed without employees or unpaid family worker).

Table 2 shows a summary of the descriptive statistics of the main variables in the three samples, which for simplicity’s sake we call “bachelor 2014”, “bachelor 2019” and “master 2019”. About 6 out of 10 bachelor’s degree graduates surveyed are women, a proportion that drops to 5 out of 10 among master’s degree graduates. Around 1 in 100 interviewees in the three samples are people with disabilities. Additionally, around 15.0% of bachelor’s degree graduates and 25.0% of master’s degree graduates had studied at a private university. In relation to distribution by branches of knowledge, graduates from the area of "social sciences and law" are clearly predominant.

**Table 2:** Descriptive statistics.

Mean (standard deviation)	Bachelor 2014	Bachelor 2019	Master 2019
% women	59.7 (49.1)	57.0 (49.5)	52.6 (49.9)
% with disability	0.9 (9.5)	1.2 (11.0)	1.0 (10.0)
% with general grant	34.6 (47.6)	38.2 (48.6)	23.2 (42.2)
% with excellence scholarship	2.3 (14.8)	4.4 (20.5)	2.2 (14.6)
% private univesity	14.0 (34.7)	14.8 (35.5)	25.2 (43.4)
% postgraduate education: master	33.0 (47.0)	47.5 (49.9)	31.6 (46.5)
% Arts and Humanities	10.6 (30.8)	10.0 (30.1)	11.0 (31.3)
% Science	9.7 (29.6)	8.8 (28.3)	10.9 (31.2)
% Social Sciences and Law	44.3 (49.7)	45.8 (49.8)	47.7 (49.9)
% Engineering and Architecture	22.4 (41.7)	21.2 (40.9)	16.7 (37.3)
% Health Sciences	13.0 (33.6)	14.2 (34.9)	13.7 (34.4)
% inactive (4–5 years later)	6.7 (25.1)	6.6 (24.8)	5.4 (22.5)
% unemployed (4–5 years later)	19.9 (40.0)	8.2 (27.5)	7.2 (25.9)
% temporary contract (4–5 years later)	34.8 (47.6)	27,9 (44.9)	29.6 (45.6)
% low wage (4–5 years later)	19.9 (39.9)	16.2 (36.9)	12.5 (33.0)
% overqualified (4–5 years later)	25.4 (43.5)	21.3 (41.0)	13.2 (33.8)
% horizontal mismatch (4–5 years later)	23.9 (42.6)	26.2 (44.0)	39.6 (48.9)
% dissatisfied (university or degree)	41.8 (49.3)	38.5 (48.7)	34.1 (47.4)
number of observations	30,379	31,651	11,483

Source: University Graduate Job Placement Survey 2014 and 2019 (INE). Own calculations.

Note: The primary data used in the empirical chapter have been extracted from the information provided by the Spanish National Institute of Statistics (INE). However, the degree of accuracy or reliability of the tables and graphs made by the author is his sole responsibility.

On the topic of scholarships, the survey provides information on "general grants" and "excellence scholarships". The main difference between these two types of scholarships is that the former are conditional on the student passing a minimum number of annual credits and their family income level not exceeding a certain threshold (for example, an income of 21,054.00 euros in four-member families in 2021) [45], while the latter are aimed at "students with an excellent level of academic achievement", regardless of household income. Specifically, in order to be eligible for an excellence scholarship, beneficiaries must surpass the following minimum GPAs, on a 0–10 scale: 8.15 points in Engineering and Architecture degrees, 8.65 in Medicine studies and 9.15 points in other degrees [46]. Therefore, general grants are a proxy variable for family socioeconomic status, while excellence scholarships are a proxy for graduates’ ability. The information in Table 2 indicates that the proportion of graduates with general grants increased from 34.6% in 2014 to 38.2% in 2019. Among master’s degree graduates, the percentage of students with general grants practically halved (23.2%). On the other hand, the percentage of undergraduate graduates receiving excellence scholarships increased from 2.3% in 2014 to 4.4% in 2019, a figure that decreased to 2.2% among master’s degree graduates.

The EILU looks at whether the interviewee completed a master’s degree in addition to the university studies for which they were selected to participate in the survey. In the period under study, study plans in Spain were reformed within the framework of the Bologna process, which led to the gradual disappearance of first- (3 years) and second-cycle (5 years) studies and to the progressive implementation of degree (4 years) and master’s (1 to 2 years) studies. Ministry of Education and Vocational Training statistics [47] reveal a very significant growth in the number of master’s degree students in Spain, from 16,609 students in the 2006/07 academic year to 81,485 in 2009/10; 122,882 in 2013/14; and, finally, 248,460 in the 2020/21 academic year. This trend is also reflected in Table 2, where the proportion of undergraduates who had completed a master’s degree as well rose from 33.0% in 2014 to 47.5% in 2019. On the other hand, in the 2019 sample of master’s degree graduates, it is striking that 31.6% of the total stated that they had completed other additional master’s studies.

In 2014, 41.8% of graduates stated that, if they had to start over, they would not study at university at all, or they would not study the same degree again. In 2019, dissatisfaction with university education decreased to 38.5% among bachelor’s degree graduates and to 34.1% among master’s degree graduates. At first glance, this reduction in dissatisfaction with university education is associated with an improvement in job placement outcomes for university graduates. Regarding graduates’ employment situation four or five years after finishing their university studies, it is observed that graduates included in the first edition of the survey (2014) entered the labor market in a context of serious economic crisis characterized by high levels of unemployment (19.9%), temporary contracts (34.8%) and overqualification (25.4%). The improvement of the economic situation by the time of the second edition of the survey (2019) led to a significant reduction in unemployment (8.2%), temporary contracts (27.9%) and overqualification (21.3%). With regard to master’s degree graduates, their labor market outcomes improved compared to those of bachelor’s degree graduates, with the exception of variables measuring temporary employment and horizontal mismatch.

These levels of dissatisfaction are, on the other hand, very similar to those obtained in other studies. For example, ANECA [22] investigates the transition into the labor market of university graduates in Spain in 2000. When asked "Looking back, if I were free to choose again, to what extent would it be very likely, quite likely, quite unlikely or unlikely that I would choose the same degree?", 30% of those surveyed answered that it would be quite unlikely or unlikely. Similarly, Michavila et al. [48] find that 27% of graduates would choose a different degree if they could go back ―a figure significantly higher than the 6% of graduates who would not pursue university studies again. Finally, in the REFLEX project, Spain once again appeared as one of the countries with lowest overall graduate satisfaction in relation to their university studies. According to Carot et al. [16], 9% of respondents answered that they would not study at university at all, while 29% indicated that they would have preferred to study a different degree.

**Fig 1:**
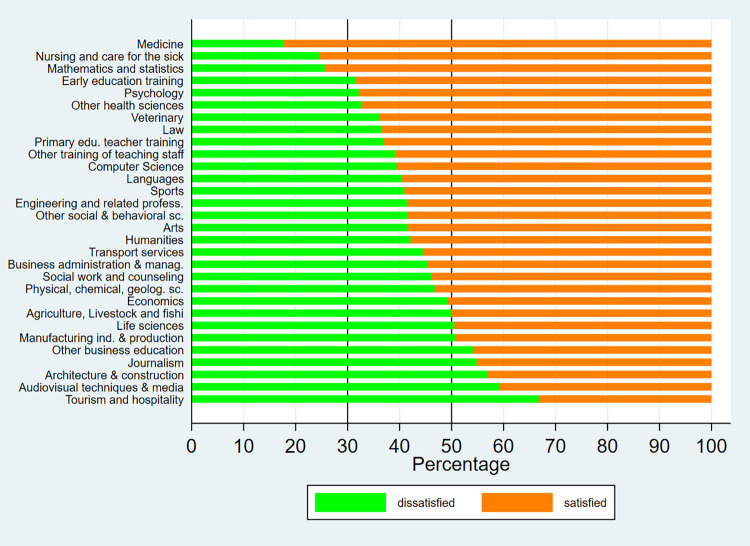
Satisfaction with university education by fields of study (Bachelor—2014). Source: University Graduate Job Placement Survey 2014 (INE). Own calculations.

**Fig 2:**
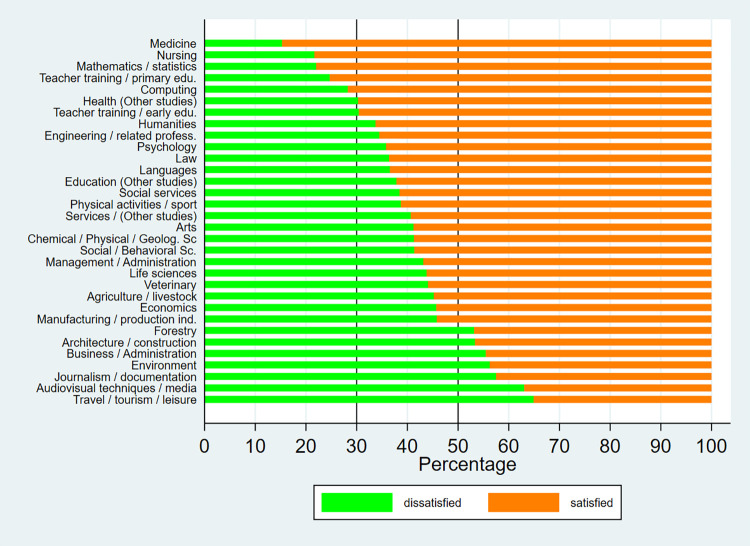
Satisfaction with university education by fields of study (Bachelor—2019). Source: University Graduate Job Placement Survey 2019 (INE). Own calculations.

**Fig 3:**
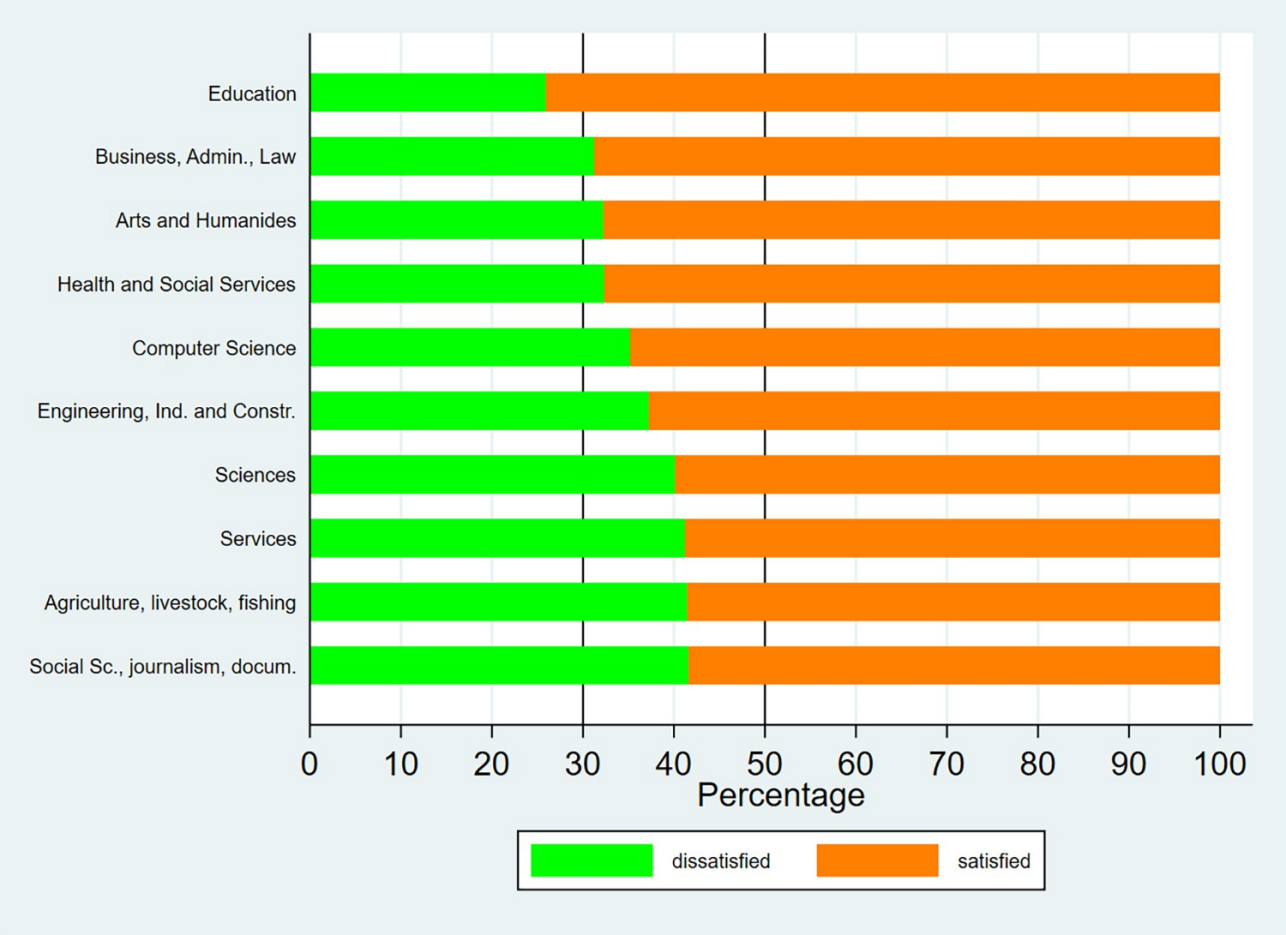
Satisfaction with university education by fields of study (Master—2019). Source: University Graduate Job Placement Survey 2019 (INE). Own calculations.

Figs 1–3 show the distribution by field of study of university graduate satisfaction. From the graphs, it can be deduced that satisfaction with received education varies notably by discipline. Among bachelor’s degree graduates, the highest level of satisfaction is registered in the fields of “medicine”, “nursing”, and “mathematics and statistics”, where over 70% of graduates are satisfied with the education they received, both in 2014 and 2019. On the other hand, the least positive outcomes are observed in “architecture”, “journalism”, “audiovisual techniques and media”, “other business education” and “travel, tourism and leisure”, where under 50% of graduates are satisfied. Regarding master’s degree graduates, over 50% of graduates across all areas are satisfied with their university education. The highest levels of satisfaction are found in the fields of “education”, “business, administration and law”, “arts and humanities”, “health and social services” and “computer science”, where more than 2/3 of graduates are satisfied with the education they received.

### Econometric analysis: Propensity-score matching

The propensity-score matching (PSM) estimator is a technique used to evaluate treatment effects using observational data [49]. The objective of this article is to measure the association between job placement results and satisfaction with university education. For example, we would like to know whether holding a job that does not match the graduate’s education level increases their dissatisfaction with university education. In order to estimate the treatment effect, we need to overcome the evaluation problem that arises from the fact that only one potential outcome is observed. The purpose of the PSM technique is to estimate the counterfactual outcome, i.e. what would the satisfaction with university education of an overeducated graduate have been in the event of them having a job matching their education level.

The difference in mean satisfaction between individuals under treatment (overqualified graduates) and the control group (graduates holding an occupation adequate to their education level) does not depend solely on the presence or absence of treatment, since overqualification is not assigned randomly among university graduates, but, rather, it is correlated with the student’s ability, the socioeconomic status of their family, or, among other variables, with the degree they completed. In addition, satisfaction with university education also depends on the student’s ability, their socioeconomic status and their field of study. Having said that, the propensity-score matching estimator selects a group of comparison consisting of subjects who are not overeducated, but with similar characteristics to the overqualified graduates in terms of gender, age, nationality, general grants, excellence scholarships, disability, postgraduate training, private university attendance, region where the university is located and field of study. In order to determine how similar subjects are to each other, PSM estimates propensity scores that represent “the conditional probability of assignment to a particular treatment given a vector of observed covariates” [50].

“ATET” stands for the average treatment effect on the treated, that is, not on the whole sample of graduates, but on those who are overqualified for their current job (T_i_ = 1). Y_1i_ represents the outcome under the treatment; in our case, the satisfaction of overqualified graduates with university education. Y_0i_ represents what the outcome would have been in the absence of treatment, that is, the satisfaction of overqualified graduates on the hypothetical assumption that they held a position adequate to their education level. The ATET is defined as:

$$ATET=E[Y_{1i}-Y_{0i}|T_{i}=1]=E[Y_{1i}|T_{i}=1]-E[Y_{0i}|T_{i}=1]$$ (1)

The problem is that, when working with observational data, we observe both the satisfaction outcomes of overqualified graduates (under treatment) and the satisfaction outcomes of graduates in occupations adequate to their education (without treatment), and the difference between the two is the average treatment effect on the treated plus the bias. The bias represents the difference between the outcomes that would be observed for treated graduates (T_i_ = 1) and controls (T_i_ = 0) in the absence of treatment (Y_0i_). It can be expressed as follows [51]:

$$E[Y_{1i}|T_{i}=1]-E[Y_{0i}|T_{i}=0]=ATET+\{E[Y_{0i}|T_{i}=1]-E[Y_{0i}|T_{i}=0]\}$$ (2)

To work out the propensity scores, a probit model of overqualification (and later, of the rest of the representative variables of labor outcomes) is estimated based on the variables mentioned above. In this work, the method of pairing used was the nearest neighbor, where each subject is matched to one individual from the other treatment level. In the implementation of PSM we followed the “teffects psmatch” subcommand of STATA 15.

In order to ensure the PSM technique functions properly, the overlap between treated and controls should be broad. In addition, the conditional-independence assumption must be met. This implies that the selection of individuals for the treatment group must be based on observable characteristics only. In the event that unobservable variables influence the probability of being overqualified and which are, in turn, correlated with satisfaction, the estimates obtained will be biased. In this case, the matching techniques do not fully solve the endogeneity problem, which is why results should be interpreted with great care, especially in the case that the dependent variable is a subjective indicator, which exacerbates the role of unobservables. For all these reasons, the results obtained in this work should not be interpreted in terms of a causal relationship.

## Results

Table 3 shows marginal effects for the probability of treatment for the six representative variables of the job placement process of graduates (inactivity, unemployment, vertical mismatch, horizontal mismatch, low wages and temporary contract). The Bachelor sample of 2019 has been chosen for a more detailed presentation of results, since the 2019 edition of the survey includes more socio-demographic variables than that of 2014 and, compared to that of master’s degree graduates, has a larger sample size. The first row of the table shows the mean probability of treatment, which in the case of inactivity amounts to 5.7%. The variables that show a greater marginal effect are disability, which increases the probability of the graduate being inactive by 8.5 percentage points, and the completion of a master’s degree, which reduces it by 2.1 points. Inactivity varies markedly by region and field of study. Compared with Madrid, the probability of being inactive is between 3.4 and 4.4 percentage points higher in the Autonomous Communities of Extremadura, Andalusia, Galicia, Murcia and the Canary Islands, while in La Rioja and Catalonia, it is 2.6 and 1.2 points lower, respectively. Regarding the fields of study, the marginal effects are mostly negative and significant, given that the reference category is Law, a degree where 14.5% of graduates are inactive, as a consequence of the fact that, five years after having finished their studies, 12.6% of the graduates are “studying or preparing for public exams”.

**Table 3:** Marginal effects after probit (Bachelor 2019).

	(1)	(2)	(3)	(4)	(5)	(6)
VARIABLES	INACTIVITY	UNEMPLOYMENT	VERTICAL MISM.	HORIZONTAL MISM.	LOW WAGE	TEMPORARY C.
Pr (y = 1)	0.057	0.072	0.166	0.226	0.094	0.251
female	0.005	0.002	-0.006	-0.014	0.025***	0.026***
age 30 to 34	-0.008**	0.007*	0.047***	0.045***	0.007	-0.011
age more than 34	-0.000	0.004	0.016	0.040***	-0.026***	-0.063***
father higher edu	0.002	0.002	-0.050***	-0.015**	-0.006	-0.010*
mother higher edu	0.004	0.005	-0.022***	-0.022**	-0.005	0.006
father primary edu	-0.003	-0.008*	0.015***	-0.002	-0.001	0.016*
mother primary edu	-0.006*	0.005	-0.004	-0.006	0.012***	-0.010
general grant	0.006**	0.014***	0.029***	0.008	0.023***	0.046***
excellence scholarship	-0.009	-0.011	-0.058***	-0.050***	-0.035***	-0.039**
master	-0.021***	-0.002	-0.114***	-0.080***	-0.022***	0.051***
private university	-0.003	-0.015**	-0.041***	-0.039***	-0.029***	-0.021*
disable	0.085***	0.031*	-0.030*	-0.006	0.006	-0.039
foreign	-0.012	0.005	0.023	0.013	0.003	-0.053***
distance university	-0.004	-0.018*	0.018	0.083***	-0.004	-0.032
part-time job			0.141***	0.105***	0.559***	0.251***
national university	0.010	0.002	0.005	0.003	-0.023	0.028
Andalucía	0.039***	0.059***	0.033**	-0.010	0.056***	0.093***
Aragón	0.013	0.013	0.025*	0.003	0.009	0.076***
Asturias	0.027***	0.030**	0.026	-0.003	0.041***	0.088***
Baleares	-0.009	-0.002	0.005	-0.028	-0.029***	0.032
Canarias	0.034***	0.035***	0.041**	0.016	0.040**	0.082***
Cantabria	0.030**	0.035*	0.024	-0.012	0.013	0.104***
Castilla y León	0.008	0.013	0.010	-0.029**	0.012	0.067***
Castilla-La Mancha	0.011	0.036***	0.031	0.007	0.036**	0.066***
Cataluña	-0.012**	-0.016**	-0.029***	-0.044***	-0.024***	0.040***
C. Valenciana	0.022***	0.028***	0.025	-0.026**	0.021**	0.050***
Extremadura	0.044**	0.047***	0.055**	0.050*	0.106***	0.095***
Galicia	0.035**	0.031**	0.020	-0.012	0.031***	0.040*
Murcia	0.035***	0.024**	0.045**	-0.009	0.085***	0.066***
Navarra	-0.005	0.005	0.027	-0.026	0.013	0.095***
País Vasco	0.002	-0.002	0.027*	-0.010	-0.007	0.104***
Rioja (La)	-0.026***	-0.007	-0.046	-0.067**	-0.019	0.100***
Education (Other studies)	-0.040***	0.004**	-0.026***	-0.015***	-0.027***	0.277***
Arts	-0.004***	0.058***	0.181***	0.196***	0.052***	0.146***
Humanities	0.005***	0.072***	0.169***	0.284***	0.027***	0.236***
Languages	-0.023***	0.028***	0.002	0.096***	-0.036***	0.247***
Social / Behavioral Sc.	-0.022***	-0.009***	0.098***	0.298***	-0.022***	0.174***
Journalism / documentation	-0.045***	0.034***	0.055***	0.108***	0.006***	0.176***
Business / Administration	-0.045***	-0.001	0.046***	0.090***	-0.036***	0.055***
Life sciences	-0.032***	0.025***	0.039***	0.021***	-0.012***	0.282***
Environment	-0.039***	-0.013***	0.056***	0.188***	-0.000	0.288***
Chemical / Physical / Geolog. Sc.	-0.032***	0.006***	0.035***	0.050***	-0.021***	0.246***
Mathematics / statistics	-0.029***	-0.041***	-0.049***	-0.032***	-0.064***	0.170***
Computing	-0.058***	-0.053***	-0.070***	-0.147***	-0.079***	-0.022***
Engineering / related profess.	-0.055***	-0.044***	-0.075***	-0.066***	-0.085***	0.055***
Manufacturing / production ind.	-0.045***	-0.007***	-0.055***	0.039***	-0.060***	0.124***
Architecture / construction	-0.046***	-0.030***	-0.046***	-0.007*	-0.053***	0.131***
Agriculture / livestock	-0.040***	-0.031***	-0.003	-0.005	-0.047***	0.155***
Forestry	-0.044***	-0.025***	0.053***	0.088***	-0.034***	0.352***
Veterinary	-0.041***	-0.020***	-0.122***	-0.149***	-0.035***	0.071***
Health (Other studies)	-0.051***	-0.041***	-0.122***	-0.122***	-0.048***	0.043***
Social services	-0.041***	-0.017***	0.007	-0.016***	-0.032***	0.274***
Services / (Other studies)	-0.047***	-0.037***	-0.031***	0.010	-0.063***	0.181***
Teacher training / early edu.	-0.037***	0.046***	0.020***	-0.021***	0.004	0.348***
Teacher training / primary edu.	-0.040***	0.019***	-0.071***	-0.072***	-0.049***	0.348***
Audiovisual techniques / media	-0.043***	0.037***	0.158***	0.184***	0.005***	0.207***
Economics	-0.036***	-0.023***	0.044***	0.023***	-0.034***	0.020***
Psychology	-0.031***	0.016***	0.024***	0.039***	-0.007***	0.161***
Management / Administration	-0.042***	-0.016***	0.046***	-0.002	-0.044***	0.035***
Medicine	-0.053***	-0.059***	-0.172***	-0.235***	-0.097***	0.448***
Nursing	-0.049***	-0.054***	-0.162***	-0.196***	-0.083***	0.481***
Physical activities / sport	-0.031***	-0.016***	0.086***	0.054***	0.005	0.208***
Travel / tourism / leisure	-0.032***	0.010***	0.172***	0.147***	-0.020***	0.176***
Observations	31,651	29,562	26,917	26,901	26,385	27,124
Pseudo R^2^	0.053	0.053	0.127	0.095	0.334	0.117

Robust standard errors in parentheses

*** p<0.01

** p<0.05

* p<0.1

Source: University Graduate Job Placement Survey 2019 (INE). Own calculations.

In relation to the rest of the columns of Table 3, the results of labor insertion of women tend to be similar to those of men, with the exception of wages, where they have a 2.5 points higher probability of receiving a low wage, and temporary employment, where the incidence of temporary contracts is 2.6 points higher. Family background, estimated through parents’ educational level, has some influence on several indicators, such as vertical and horizontal educational mismatch. For example, having a father with a higher education reduces the probability of being overqualified by 5.0 percentage points, as does having a mother with university studies, which does so by 2.2 points. Graduates with general grants, conditional on the household income, tend to have less satisfactory job placement results in terms of higher unemployment, inactivity, overqualification, low wages and temporary employment rates. On the other hand, being awarded an excellence scholarship, based only on the student’s academic performance, reduces the incidence of educational mismatch, low wages and temporary employment. Furthermore, completing a master’s degree or studying at a private university improves job placement outcomes, while working part-time worsens them.

Finally, there are significant differences in job placement results by region and, especially, by field of study. To mention a few examples, while the average probability of being overqualified for the reference category (“Law”) is 16.6%, the probability is higher in “Arts” (18.1 points higher), “Travel, tourism and leisure” (17.2 points more), “Humanities” (16.9 points higher) and “Audiovisual techniques & media” (15.8 points higher). Instead, the probability of being overqualified drops by 16.2 points in “Nursing” and by 17.2 points in “Medicine”. In order to speed up the presentation of results, the tables collecting the marginal effects for the probability of treatment of the Master (2019) and Bachelor (2014) samples have been moved to the S1 Annex.

Table 4 shows the standardized mean differences before and after matching. For each covariate, the difference in sample means between the treated subjects and the controls is calculated. The result is then divided by the square root of the average of the sample variances in both groups. Examining the standardized differences allows us to check whether the covariates are balanced over treatment levels. Calculating standardized differences is an exploratory diagnostic technique that does not include a test that could assess the success of the matching procedure. However, in empirical studies [51], a reduction of the differences below the range 0.03–0.05 is usually considered sufficient. The results in Table 4 indicate that PSM significantly reduces the differences between raw and matched data. For example, in relation to inactivity, it is observed that the differences in the means of the disability (0.142) and master (-0.084) variables tend to zero in the matched data. The same happens with the dummies related to the field of study that go, for example, from 0.163 to 0.012 in “Arts”, from 0.186 to 0.026 in “Humanities”, from -0.203 to 0.006 in “Computing” and from -0.210 to -0.008 in “Engineering and related professions”. The same reduction is observed in the regional dummies, where, for example, the difference goes from 0.139 to -0.031 in Andalusia and from -0.149 to 0.019 in Catalonia. Examining the rest of the columns allows us to check how the PSM technique improved the level of balance in the matched sample, insofar as the weighted standardized mean differences are all close to zero.

**Table 4:** Standardized mean differences before and after matching (Bachelor 2019).

*Covariate balance summary*															
BACHELOR 19	INACTIVITY		UNEMPLOYMENT		VERTICAL MISM.		HORIZONTAL MISM.			LOW WAGE			TEMPORARY C.		
	Raw	Matched	Raw	Matched	Raw	Matched	Raw		Matched	Raw	Matched		Raw		Matched
Number of obs	31,651	4,178	29,562	4,876	26,917	11,476	26,901		14,102	26,385	8,560		31,487		15,144
Treated obs	2,089	2,089	2,438	2,438	5,738	5,738	7,051		7,051	4,280	4,280		7,572		7,572
Control obs	29,562	2,089	27,124	2,438	21,179	5,738	19,850		7,051	22,105	4,280		23,915		7,572
*Standardized differences*															
	INACTIVITY		UNEMPLOYMENT		VERTICAL MISM.		HORIZONTAL MISM.			LOW WAGE				TEMPORARY C.	
covariates	Raw	Matched	Raw	Matched	Raw	Matched	Raw	Matched		Raw		Matched		Raw	Matched
mother primary edu	-0.029	0.022	0.03	0.03	0.15	0.02	0.09	0.01		0.05		0.05		-0.03	0.01
mother higher edu	0.011	-0.006	-0.03	0.02	-0.25	0.03	-0.14	0.00		-0.14		-0.02		-0.03	0.02
father primary edu	-0.023	0.018	0.00	0.02	0.17	0.02	0.08	0.01		0.07		0.05		0.02	0.01
father higher edu	-0.001	0.023	-0.05	-0.01	-0.30	0.02	-0.13	0.00		-0.17		0.00		-0.07	0.03
disable	0.142	0.000	0.04	0.05	-0.01	0.00	0.01	0.01		0.01		-0.01		-0.03	0.00
female	0.084	-0.057	0.10	-0.02	0.02	0.01	-0.04	-0.01		0.30		-0.01		0.22	-0.04
age 30 to 34	-0.076	-0.001	0.02	0.03	0.15	0.01	0.08	0.01		0.06		0.02		-0.02	0.03
age more than 34	-0.015	0.054	-0.09	0.02	0.04	-0.03	0.12	0.00		-0.21		-0.01		-0.20	0.00
foreign	-0.032	0.027	0.01	0.03	0.02	0.01	0.01	-0.01		0.00		0.00		-0.04	-0.01
general grant	0.102	0.007	0.19	-0.04	0.20	-0.01	0.05	0.02		0.31		0.03		0.18	-0.01
excellence scholarship	-0.042	0.016	-0.05	0.01	-0.14	0.04	-0.11	0.03		-0.11		0.03		-0.01	0.02
private university	-0.129	-0.045	-0.17	0.03	-0.18	0.00	-0.11	0.01		-0.18		-0.02		-0.05	-0.01
distance university	-0.036	-0.021	-0.11	0.01	0.04	-0.05	0.16	-0.01		-0.14		0.02		-0.12	0.02
master	-0.084	0.002	0.03	0.01	-0.36	-0.01	-0.17	-0.02		-0.02		-0.05		0.13	0.01
part-time job					0.34	0.04	0.22	0.01		1.40		0.00		0.55	0.02
Education (Other studies)	0.011	-0.005	0.06	0.01	0.05	0.00	0.03	0.00		0.14		-0.01		0.09	0.00
Arts	0.163	0.012	0.14	0.01	0.17	-0.01	0.15	-0.01		0.18		-0.05		-0.03	0.02
Humanities	0.186	0.026	0.16	0.01	0.15	0.01	0.21	0.01		0.15		-0.04		0.01	0.03
Languages	0.080	0.009	0.10	0.02	-0.01	0.00	0.08	0.00		0.10		-0.01		0.07	0.01
Social / Behavioral Sc.	0.073	0.022	-0.01	0.03	0.10	0.01	0.21	-0.02		0.02		-0.01		-0.02	0.02
Journalism / documentation	-0.060	0.011	0.08	0.00	0.04	0.01	0.07	0.01		0.05		-0.02		-0.01	0.01
Business / Administration	-0.059	0.015	0.01	0.02	0.05	-0.02	0.07	0.01		-0.03		0.04		-0.09	0.00
Life sciences	0.011	0.050	0.08	-0.01	-0.01	0.02	-0.03	0.01		0.02		0.01		0.07	0.00
Environment	-0.018	0.013	-0.01	0.00	0.05	0.00	0.12	0.00		0.06		0.01		0.06	-0.01
Chemical / Physical / Geolog. Sc.	0.019	0.008	0.04	-0.01	0.01	0.02	0.02	0.02		0.01		0.03		0.04	0.00
Mathematics / statistics	0.027	0.048	-0.07	-0.02	-0.06	0.04	-0.04	0.01		-0.09		-0.01		-0.01	0.00
Computing	-0.203	0.006	-0.17	0.00	-0.07	-0.01	-0.17	0.01		-0.23		0.00		-0.18	0.01
Engineering / related profess.	-0.210	-0.008	-0.20	0.00	-0.17	0.00	-0.13	-0.02		-0.36		0.03		-0.18	-0.01
Manufacturing / production ind.	-0.055	-0.025	0.00	0.00	-0.05	0.01	0.03	-0.01		-0.09		0.02		-0.04	-0.01
Architecture / construction	-0.084	-0.011	-0.09	0.00	-0.06	0.00	-0.01	-0.01		-0.13		0.04		-0.07	-0.01
Agriculture / livestock	-0.012	0.024	-0.04	0.05	0.03	0.00	0.01	-0.01		-0.05		0.01		-0.03	0.03
Forestry	-0.028	0.024	-0.02	0.00	0.05	0.02	0.06	-0.01		-0.01		-0.03		0.05	0.01
Veterinary	-0.013	-0.013	-0.02	-0.03	-0.11	-0.02	-0.11	-0.01		0.00		-0.05		-0.05	0.02
Health (Other studies)	-0.121	0.003	-0.13	-0.01	-0.21	0.02	-0.17	-0.01		0.02		-0.04		-0.08	0.01
Social services	-0.022	0.003	-0.03	-0.02	0.06	-0.01	0.01	0.02		0.06		0.06		0.07	-0.02
Services / (Other studies)	-0.038	0.024	-0.04	-0.01	-0.02	0.00	0.01	0.01		-0.08		0.00		-0.01	-0.01
Teacher training / early edu.	0.027	-0.013	0.16	-0.01	0.11	-0.01	0.01	0.01		0.21		0.04		0.13	-0.02
Teacher training / primary edu.	-0.008	-0.028	0.09	0.00	-0.08	0.03	-0.08	0.02		0.05		0.00		0.15	-0.02
Audiovisual techniques / media	-0.038	-0.036	0.07	0.04	0.11	-0.01	0.10	0.00		0.08		0.00		0.02	0.01
Economics	0.002	0.009	-0.04	-0.02	0.05	0.00	0.02	0.00		-0.05		-0.01		-0.10	0.00
Psychology	0.028	-0.020	0.04	-0.02	0.03	-0.04	0.04	0.00		0.13		0.01		0.01	-0.01
Management / Administration	-0.025	-0.020	-0.04	-0.02	0.12	0.01	0.01	-0.01		-0.09		0.05		-0.18	0.00
Medicine	-0.111	0.000	-0.16	-0.01	-0.25	-0.01	-0.26	0.00		-0.23		-0.07		0.18	0.00
Nursing	-0.089	-0.012	-0.17	0.01	-0.28	-0.02	-0.27	0.01		-0.14		0.01		0.28	-0.01
Physical activities / sport	0.026	-0.022	-0.02	0.00	0.09	-0.03	0.04	0.00		0.16		0.00		0.05	0.00
Travel / tourism / leisure	0.037	0.000	0.03	-0.01	0.16	-0.03	0.11	0.00		0.03		-0.04		-0.02	-0.01
National university	0.019	-0.010	-0.07	0.00	0.09	-0.06	0.18	-0.02		-0.09		0.03		-0.12	0.01
Andalucía	0.139	-0.031	0.20	-0.03	0.08	-0.01	0.01	-0.03		0.16		0.07		0.05	-0.03
Aragón	-0.006	-0.002	-0.01	-0.01	0.00	0.01	0.00	0.03		-0.01		0.03		0.03	-0.02
Asturias	0.043	0.020	0.04	-0.02	0.02	0.03	0.00	0.00		0.04		-0.06		0.03	0.00
Baleares	-0.046	0.015	-0.01	0.00	0.01	-0.02	-0.01	0.00		-0.04		-0.03		0.00	0.01
Canarias	0.065	0.000	0.06	-0.01	0.05	-0.01	0.03	0.01		0.04		-0.06		0.02	0.02
Cantabria	0.024	0.049	0.01	0.01	-0.02	0.02	-0.03	0.00		-0.03		0.01		0.02	0.01
Castilla y León	-0.042	0.007	-0.02	0.01	-0.02	0.03	-0.03	-0.01		-0.02		0.04		0.02	0.03
Castilla-La Mancha	-0.002	0.039	0.05	0.04	0.03	-0.01	0.01	0.03		0.06		-0.03		0.03	-0.01
Cataluña	-0.149	0.019	-0.17	0.01	-0.11	-0.02	-0.05	0.00		-0.13		0.00		-0.02	-0.01
C. Valenciana	0.028	-0.013	0.04	0.01	0.04	-0.02	-0.01	0.03		0.03		0.00		-0.01	0.01
Extremadura	0.063	0.002	0.07	-0.01	0.04	0.04	0.04	0.02		0.10		-0.03		0.04	0.00
Galicia	0.077	-0.024	0.05	0.02	0.02	0.01	0.00	-0.04		0.05		-0.02		0.00	-0.01
Murcia	0.069	0.014	0.02	0.01	0.04	0.01	0.00	0.03		0.12		0.04		0.02	0.00
Navarra	-0.064	-0.011	-0.06	0.01	-0.05	-0.02	-0.07	0.00		-0.07		-0.01		0.03	-0.03
País Vasco	-0.052	-0.030	-0.07	0.03	0.00	0.00	0.00	0.00		-0.02		-0.04		0.06	0.03
Rioja (La)	-0.092	-0.010	-0.06	0.04	-0.04	-0.02	-0.02	0.00		-0.05		0.02		0.02	0.06

Source: University Graduate Job Placement Survey 2019 (INE). Own calculations.

As noted above, in order to apply the PSM method, there needs to be a sufficient overlap between the individuals under treatment and the control group. In particular, in order to estimate the ATET it is necessary to check the existence of potential matches in the control group. A1 to A18 Figs in S1 Annex show the density function of the propensity scores in both groups before and after the matching for all six different treatment models and using the three samples from the EILTU Survey (INE). By examining the graphs, it can be concluded that there is no evidence that the overlap assumption is breached, since the common support region is sufficiently wide. The overlap of the distributions of the treated and control groups in the plots using the matched data is particularly striking.

Table 5 shows estimates of the average treatment effect on the treated (ATET) in relation to the six representative variables of the graduates’ job placement process. Panel A shows the results for the 2014 bachelor’s degree sample; panel B, those relating to the 2019 bachelor’s degree sample; and panel C, those using the sample of master’s degree graduates in 2019. First of all, it should be noted that, compared to the rest of the treatment variables, the two indicators that show the highest ATET are vertical and horizontal educational mismatch. In the “Bachelor 2019” sample, both being overqualified and working outside one’s area of study increase dissatisfaction with university studies by 25.0 percentage points. Vertical mismatch increases dissatisfaction in 26.1 points in the “Master 2019” sample, while the influence of horizontal mismatch is slightly lower at 21.7 points. Finally, in the "Bachelor 2014" sample, the ATETs of both horizontal and vertical mismatch exceed 20 percentage points. In conclusion, results indicate that carrying out a job that requires a lower education level or that is outside one’s own field of study has a very notable influence on graduates’ dissatisfaction with their university experience. This result is in line with García-Aracil [11], who found, as noted above, that overqualified graduates, as well as those who work outside their field of study, are less satisfied with their education.

**Table 5:** Estimates of the effect of job placement results on dissatisfaction with university education.

Panel A–Bachelor (2014)					
variables	coefficient	robust s.e.	z	P>|z|	observations
Inactivity	0.036	0.013	2.840	0.004	30,379
Unemployment	0.116	0.009	12.650	0.000	28,331
Vertical mismatch	0.202	0.010	19.470	0.000	21,540
Horizontal mismatch	0.213	0.011	19.910	0.000	21,426
Low wage	0.089	0.013	6.710	0.000	17,777
Temporary contract	-0.002	0.010	-0.230	0.817	22,214
Panel B–Bachelor (2019)					
variables	coefficient	robust s.e.	z	P>|z|	observations
Inactivity	0.090	0.014	6.250	0.000	31,651
Unemployment	0.137	0.013	10.210	0.000	29,562
Vertical mismatch	0.250	0.010	23.780	0.000	26,917
Horizontal mismatch	0.250	0.009	26.910	0.000	26,901
Low wage	0.111	0.017	6.600	0.000	26,385
Temporary contract	-0.003	0.009	-0.320	0.750	27,124
Panel C–Master (2019)					
variables	coefficient	robust s.e.	z	P>|z|	observations
Inactivity	0.059	0.027	2.160	0.031	10,630
Unemployment	0.138	0.025	5.560	0.000	10,077
Vertical mismatch	0.261	0.020	13.270	0.000	9,307
Horizontal mismatch	0.217	0.013	16.890	0.000	9,289
Low wage	0.153	0.029	5.310	0.000	9,119
Temporary contract	0.047	0.016	3.040	0.002	9,363

Source: University Graduate Job Placement Survey 2014 and 2019 (INE). Own calculations.

On the other hand, the comparison of the results of panels A (2014) and B (2019) of Table 5 shows a notable growth of the coefficients in the year 2019 compared to 2014. Especially in the case of inactivity, vertical and horizontal mismatch, and to a lesser extent in the case of unemployment, the coefficients tend to increase around 4 to 5 percentage points in 2019, when the youth unemployment rates in Spain had been reduced by around 20 percentage points compared to 2014, one of the worst years of the Great Recession. This result could be explained by the improvement in the expectations of graduates after 5 years of employment growth. When the economic situation improves, job placement problems increase dissatisfaction with the university experience to a greater extent than in periods of economic crisis, when graduates’ expectations are lower in relation to the probability of finding a good job.

The reduction in the influence of horizontal mismatch on university satisfaction among master’s degree graduates compared to bachelor’s degree graduates in 2019 could be explained by the lower incidence of involuntary horizontal mismatch among the former. Among bachelor’s degree graduates, 56.3% of those who worked outside their field of study also stated that they were overqualified, in contrast to master’s degree graduates, where the proportion was around half (27.7%). Empirical evidence suggests that, while involuntary horizontal mismatch is associated with an earnings penalty, working outside your field of study as a result of good knowledge of job opportunities in other areas is associated with an increase in wages [38].

Unemployment and earning low wages are the other two indicators that show a substantial and statistically significant association with dissatisfaction. The ATET for unemployment is between 11.6 points in the “Bachelor 2014” sample and 13.8 points in the “Master 2019” sample. On the other hand, the association between dissatisfaction and earning low wages in 2019 is higher among master’s degree graduates (15.3 percentage points) than bachelor’s degree graduates (11.1 points). When interpreting this result, the higher salary expectations of graduate students compared to undergraduate students should be taken into account. On the other hand, earning low wages increases the dissatisfaction of 2014 graduates by 8.9 percentage points. As pointed out above, this result is not strictly comparable with those of 2019, since the definition of the wage variable changed in the second wave of the survey. Finally, it should be noted that the results obtained in relation to the association on satisfaction with the indicators of educational mismatch, wages and unemployment are consistent with those obtained by Whelan and McGuinness [14].

Inactivity shows a positive and statistically significant relationship, although smaller in size, with the dissatisfaction of graduates with their university experience. The ATET among bachelor’s degree graduates varies between 3.6 percentage points in 2014 and 9.0 in 2019. The interpretation of these results requires more detailed analysis, since the inactive category includes graduates who temporarily leave the labor market due to difficulties finding a satisfactory job (pursuing other studies, preparing competitive exams and people dedicated to housework) along with graduates who withdraw from the labor market permanently (retired or unable to work).

In order to explore the association between dissatisfaction and the different situations of inactivity identified in the survey, a linear model of the determinants of university dissatisfaction has been estimated (see Table 6). The first situation analyzed is that of graduates who are still studying or preparing for public examinations five years after finishing their studies. The second one comprises university graduates dedicated to housework. The third one includes graduates who are retired or unable to work. As can be seen in the table, the first two situations present a positive and mostly significant association with dissatisfaction with the university experience. On the other hand, the third one presents the opposite sign for all three estimates, showing a significant relationship in the samples of bachelor’s graduates in 2014 and 2019, and a marginally not significant one in that of master’s degree graduates in 2019. The results indicate that retired and disabled graduates, whose motivation to study has more to do with personal satisfaction than with the expectation of developing a professional career, are more satisfied with their experience at university.

**Table 6:** Association between different situations of inactivity and dissatisfaction with university education.

	Bachelor 2014			Bachelor 2019			Master 2019		
variables	(1)	(2)	(3)	(4)	(5)	(6)	(7)	(8)	(9)
student—prep. competition exams	0.033			0.078***			0.076***		
housework		0.057			0.141***			0.149**	
retired & incapacitated			-0.161***			-0.069*			-0.096
Constant	0.418***	0.422***	0.422***	0.384***	0.394***	0.395***	0.297***	0.300***	0.300***
Observations	30,379	30,379	30,379	31,651	31,651	31,651	10,630	10,630	10,630
R-squared	0.063	0.063	0.064	0.065	0.064	0.064	0.027	0.027	0.026

Note: Controls of the personal characteristics of the graduates, their educational process, family background, field of study and region are included.

Robust standard errors in parentheses

*** p<0.01

** p<0.05

* p<0.1

Source: University Graduate Job Placement Survey 2014 and 2019 (INE). Own calculations.

The last indicator of labor insertion studied in Table 5 is temporary employment. Analysis results indicate that having a temporary contract has no influence on dissatisfaction with the university experience in the 2014 and 2019 samples of bachelor’s degree, while among the 2019 master’s degree graduates the ATET is small (4.7 percentage points) but statistically significant. The choice of indicator is based on the fact that, for many experts, excessive temporality is one of the main dysfunctions of the Spanish labor market, to the extent that it produces an increase in job rotation and income inequality, it is one of the factors delaying young people’s emancipation, it has a long-term negative influence on the work trajectories of low-skilled youth [52], it negatively affects the probability of receiving on-the-job training [53] and, among other effects, it reduces labor productivity [54].

An examination of Eurostat data confirms the high share of temporary jobs in Spain. In 2019, the temporary employment rate was 11.7% in the EU-27, 9.3% in Germany, 12.1% in France, 13.1% in Italy and 21.9% in Spain. This problem affects young people in particular, since, according to the Spanish Labor Force Survey (INE), while 26.3% of the total salaried population had a temporary contract in 2019, the proportion increases to 69.5% among young people aged 16 to 24. The high percentage of temporary employment translates into excessive job turnover which, in turn, is reflected on the fact that, according to 2019 State Public Employment Service data, young people between 16 and 25 years old sign 4.4 annual contracts per employee on average, a ratio that quadruples that of the general population (1.1 contracts per employee). Taking into account all of the above, it seems logical that, when young people are asked “*In your opinion*, *what are the two aspects that make a job good*?*”* 56.5% of young people state “that it is a secure job (stable/permanent)” as their priority, and secondly, “that it is well paid” (38.2% of the total) [55].

In summary, the results of the PSM estimation indicate that the two job placement indicators with the greatest influence on graduates’ satisfaction with their university experience are vertical and horizontal educational mismatch. The fact that university graduates are forced to work in lower-skilled jobs or outside their field of studies has a significant negative relationship with their satisfaction with the education they received. Likewise, although to a lesser extent, not having a job, earning a low wage or being inactive five years after graduation, increases graduates’ dissatisfaction. On the other hand, for most graduates, having a temporary contract does not influence satisfaction with university studies, a somewhat surprising result, which could be explained either because they trust that, as their experience increases, their temporary contract will become permanent, or because they believe that the problem is not the university system’s responsibility and that it is due to the poor design of the institutions regulating the labor market in Spain. In this sense, temporary employment can negatively affect graduates’ job satisfaction and have no influence on their satisfaction with university education.

In order to evaluate the robustness of the PSM estimation results, the models have been re-estimated with different matching algorithms and using other alternative treatment effect estimators (TEs). For example, in the estimates collected in Table 5, it was established by default that the minimum number of matches per observation in the treatment group was 1. In Table 7 the same results are replicated, establishing that said minimum increases to 5 and 10 matches per observation. As can be seen in columns PSM (NN = 5) and PSM (NN = 10), results are very stable and they only experience minor variations, which, in the case of the 2014 and 2019 Bachelor’s degree samples do not exceed 1.0 percentage point. Small exceptions are observed in the 2019 master’s degree graduates sample, which, as noted above, has fewer observations. For example, if the number of matches rises to 5, the unemployment ATET increases by 2.5 percentage points, the temporary contract ATET decreases by 1.9 points and the low wage ATET decreases by 1.5 points. When the minimum number of matches is raised to 10, the ATET for unemployment increases by 2.0 points, the ATET for temporary employment decreases by 2.7 points and the ATET for low wages does so by 1.6 points. In all these cases, the estimated coefficients remain statistically significant.

**Table 7:** Estimates of the association between job placement results and graduate dissatisfaction with different matching algorithms or TE estimators.

Panel A–Bachelor (2014)	NN (5)		NN (10)		IPW		NNMATCH Mahalanobis		NNMATCH metric: Euclidean	
variables	coeff.	rob. s.e.	coeff.	rob. s.e.	coeff.	rob. s.e.	coeff.	rob. s.e.	coeff.	rob. s.e.
Inactivity	0.036***	0.012	0.038***	0.012	0.034***	0.011	0.039***	0.013	0.037***	0.012
Unemployment	0.128***	0.008	0.128***	0.008	0.130***	0.007	0.113***	0.009	0.114***	0.009
Vertical mismatch	0.202***	0.009	0.206***	0.009	0.204***	0.008	0.198***	0.010	0.198***	0.010
Horizontal mismatch	0.218***	0.009	0.219***	0.009	0.213***	0.008	0.214***	0.011	0.217***	0.010
Low wage	0.084***	0.011	0.083***	0.011	0.085***	0.010	0.074***	0.013	0.077***	0.012
Temporary contract	0.005	0.008	0.005	0.008	0.007	0.008	0.011	0.009	0.014	0.009
Panel B–Bachelor (2019)	NN (5)		NN (10)		IPW		NNMATCH Mahalanobis		NNMATCH metric: Euclidean	
variables	coeff.	rob. s.e.	coeff.	rob. s.e.	coeff.	rob. s.e.	coeff.	rob. s.e.	coeff.	rob. s.e.
Inactivity	0.080***	0.012	0.081***	0.012	0.078***	0.011	0.084***	0.014	0.081***	0.013
Unemployment	0.137***	0.011	0.138***	0.011	0.137***	0.010	0.127***	0.013	0.135***	0.012
Vertical mismatch	0.251***	0.009	0.251***	0.008	0.251***	0.008	0.257***	0.010	0.255***	0.009
Horizontal mismatch	0.244***	0.008	0.241***	0.008	0.243***	0.007	0.250***	0.009	0.248***	0.008
Low wage	0.116***	0.013	0.118***	0.013	0.112***	0.013	0.112***	0.012	0.114***	0.011
Temporary contract	0.000	0.008	0.003	0.007	0.001	0.007	0.010	0.009	0.007	0.008
Panel C–Master (2019)	NN (5)		NN (10)		IPW		NNMATCH Mahalanobis		NNMATCH metric: Euclidean	
variables	coeff.	rob. s.e.	coeff.	rob. s.e.	coeff.	rob. s.e.	coeff.	rob. s.e.	coeff.	rob. s.e.
Inactivity	0.067***	0.022	0.069***	0.021	0.069***	0.022	0.071***	0.028	0.068***	0.026
Unemployment	0.163***	0.020	0.158***	0.020	0.154***	0.019	0.134***	0.025	0.143***	0.023
Vertical mismatch	0.263***	0.016	0.267***	0.015	0.264***	0.015	0.261***	0.020	0.281***	0.018
Horizontal mismatch	0.207***	0.011	0.209***	0.010	0.204***	0.010	0.207***	0.013	0.216***	0.012
Low wage	0.138***	0.023	0.137***	0.023	0.132***	0.021	0.135***	0.022	0.148***	0.020
Temporary contract	0.028**	0.013	0.020*	0.012	0.020*	0.012	0.036**	0.015	0.038***	0.013

Robust standard errors in parentheses

*** p<0.01

** p<0.05

* p<0.1

Source: University Graduate Job Placement Survey 2014 and 2019 (INE). Own calculations.

The next column shows the results of using the inverse-probability weighting (IPW) estimator. This estimator gauges the treatment effects in two stages: in the first one, it calculates IPW weights, which represent the inverse of the probability of participating in the treatment; in a second stage, said weights are used to compute the weighted averages of the outcomes for each treatment level. The general pattern of the results obtained using the IPW estimator is very similar to that obtained using PSM. In the Bachelor samples, the most notable variations are the 1.4-point increase in the ATET of unemployment in 2014 and the 1.2-point reduction in the ATET of inactivity in 2019. Again, the changes are slightly more pronounced in the 2019 sample of Master’s degree graduates, and the greatest variation is registered for the ATET of temporary contracts, which falls by 2.7 points, although it remains statistically significant at the 10% level. Finally, the nearest-neighbor matching estimator (NNM) is used, which calculates the counterfactual by using a weighted function of the covariates for each observation. In this case, two distance metrics for covariates have been used: Mahalanobis and Euclidean. Once again, results are very stable and confirm the robustness of the estimates obtained using propensity score matching.

Finally, it should be noted that, with the aim of making the most out of the information contained in the survey, the estimates have been replicated by PSM, substituting the dummies of field of study for those of degrees, where the level of disaggregation reaches 101 categories. The results are very similar to those shown in Table 5, with a reduction of just 1.5 percentage points in the ATET for unemployment and for overqualification. The additional tables are available from the corresponding author upon request.

On the other hand, a sensitivity analysis has been carried out to check whether the inference about the estimated treatment effects can change in case of unobserved heterogeneity. In order to do this, the Mantel-Haenszel tests statistics that provide bound estimates of significance levels at given values of hidden bias [56] are calculated. We will focus on the bounds under the assumption that we overestimated the true effect of the treatment (Q_mh +), given the positive ATET in most estimations. The results in Table 8 indicate that, in most of the models, “Γ” has to be high for the ATET to be not significant at the 5% level. In the models related to educational mismatch, the estimated treatment effect is no longer significant at 5% for gamma values that oscillate between Γ = 2.700 when the treatment variable is vertical mismatch in the 2019 Bachelor sample, and Γ = 2.200 for the same variable in the 2014 Bachelor sample. The same happens in the case of horizontal mismatch, where effects are no longer significant for gamma values of Γ = 2.650 in the 2019 Bachelor sample and Γ = 2.300 in the 2014 Bachelor sample. In both models, the gammas obtained in the Master’s degree sample are in an intermediate position. Gamma values are still high for unemployment and low wages, and the lowest level is obtained for low wages in the Bachelor 2014 sample (Γ = 1.300).

**Table 8:** Rosenbaum bounds for ATET in the presence of unobserved heterogeneity.

Panel B–Bachelor (2014)					
variables	Gamma	Q_mh+	Q_mh-	p_mh+	p_mh-
Inactivity	1.050	1.466	2.906	0.071	0.002
Unemployment	1.500	1.307	19.485	0.096	0.000
Vertical mismatch	2.200	1.270	36.192	0.102	0.000
Horizontal mismatch	2.300	1.241	37.026	0.107	0.000
Low wage	1.300	0.993	10.445	0.160	0.000
Temporary contract	1.100	2.125	2.464	0.017	0.007
Panel B–Bachelor (2019)					
variables	Gamma	Q_mh+	Q_mh-	p_mh+	p_mh-
Inactivity	1.350	1.228	10.484	0.110	0.000
Unemployment	1.600	1.171	16.967	0.121	0.000
Vertical mismatch	2.700	1.468	49.616	0.071	0.000
Horizontal mismatch	2.650	1.441	52.898	0.075	0.000
Low wage	1.450	1.567	15.842	0.059	0.000
Temporary contract	1.050	1.776	0.710	0.038	0.239
Panel B–Máster (2019)					
variables	Gamma	Q_mh+	Q_mh-	p_mh+	p_mh-
Inactivity	1.050	1.284	2.049	0.100	0.020
Unemployment	1.500	1.613	8.995	0.053	0.000
Vertical mismatch	2.600	1.584	23.766	0.057	0.000
Horizontal mismatch	2.500	1.545	33.054	0.061	0.000
Low wage	1.600	1.530	10.754	0.063	0.000
Temporary contract	1.150	1.002	5.2611	0.158	0.000

Gamma: odds of differential assignment due to unobserved factors

Q_mh+: Mantel-Haenszel statistic (assumption: overestimation of treatment effect)

Q_mh-: Mantel-Haenszel statistic (assumption: underestimation of treatment effect)

p_mh+: significance level (assumption: overestimation of treatment effect)

p_mh-: significance level (assumption: underestimation of treatment effect)

Source: University Graduate Job Placement Survey 2014 and 2019 (INE). Own calculations.

As for inactivity, results should be interpreted with more caution, especially in the Bachelor 2014 and Master 2019 samples, since the confidence interval for the effect would include zero if an unobservable variable caused the odds ratio of treatment assignment to differ by 1.050 between the treatment and control groups and if this confounding variable had a substantial influence on dissatisfaction with the university experience. Likewise, estimates related to temporary contracts must be carefully analyzed since, in this case, for values of Γ = 1.050 (Bachelor 2019) or Γ = 1.100 (Bachelor 2014), treatment effects would become statistically significant at the 5% level.

The empirical section ends with an examination of heterogeneity of coefficient sizes, based on different socio-demographic and educational variables. In order not to include an unnecessarily high number of tables, we have focused on exploring heterogeneity in the Bachelor 2019 sample, with one exception mentioned below. Panel A of Table 9 shows the PSM estimates by sex and age. First, it should be noted that the general pattern of results is broadly maintained according to the first two dimensions that were investigated. The two treatment variables with the greatest association with university experience dissatisfaction are horizontal and vertical educational mismatch, regardless of the graduates’ gender and age. This first general conclusion could be qualified if one took into account that the estimated ATETs in women are slightly higher than those of men in the case of horizontal educational mismatch, low wages and, to a lesser extent, inactivity.

**Table 9:** Heterogeneity in the estimates of the association between graduate labour market outcomes and dissatisfaction (Bachelor 2019 and 2014).

	by sex				by age					
Panel A–Bachelor (2019)	Male		Female		< 30 years		30 to 34 years		> = 35 years	
variables	coeff.	rob. s.e.	coeff.	rob. s.e.	coeff.	rob. s.e.	coeff.	rob. s.e.	coeff.	rob. s.e.
Inactivity	0.053**	0.023	0.078***	0.018	0.083***	0.019	0.103***	0.029	0.077***	0.030
Unemployment	0.159***	0.023	0.115***	0.017	0.173***	0.018	0.090***	0.025	0.125***	0.030
Vertical mismatch	0.270***	0.015	0.259***	0.014	0.264***	0.016	0.210***	0.019	0.224***	0.020
Horizontal mismatch	0.219***	0.014	0.260***	0.013	0.271***	0.014	0.259***	0.018	0.158***	0.019
Low wage	0.069***	0.026	0.111***	0.020	0.089***	0.023	0.154***	0.026	0.142***	0.033
Temporary contract	0.011	0.016	0.002	0.011	0.022*	0.013	0.054***	0.019	0.051**	0.021
	by field of study									
Panel B–Bachelor (2019)	Arts & Humanities		Science		Social Sciences & Law		Engineering & Archit.		Health Sciences	
variables	coeff.	rob. s.e.	coeff.	rob. s.e.	coeff.	rob. s.e.	coeff.	rob. s.e.	coeff.	rob. s.e.
Inactivity	0.012	0.035	0.060	0.044	0.058***	0.019	0.126***	0.037	0.158***	0.035
Unemployment	0.137***	0.034	0.161***	0.042	0.141***	0.018	0.152***	0.037	0.083*	0.042
Vertical mismatch	0.249***	0.030	0.198***	0.037	0.249***	0.014	0.241***	0.022	0.333***	0.038
Horizontal mismatch	0.209***	0.027	0.215***	0.031	0.259***	0.013	0.239***	0.020	0.278***	0.031
Low wage	0.089**	0.039	0.119**	0.050	0.143***	0.024	0.091**	0.040	0.010	0.041
Temporary contract	0.037	0.031	0.012	0.026	-0.003	0.014	0.040*	0.022	0.031	0.023
	by type of university						by duration of studies			
Panel C–Bachelor (2019)	Face-to-face		Distance		Panel D–Bachelor (2014)		3 year degree		5 year degree	
variables	coeff.	rob. s.e.	coeff.	rob. s.e.	variables		coeff.	rob. s.e.	coeff.	rob. s.e.
Inactivity	0.093***	0.015	0.052	0.035	Inactivity		0.077***	0.019	0.006	0.017
Unemployment	0.148***	0.014	0.082	0.054	Unemployment		0.102***	0.013	0.138***	0.013
Vertical mismatch	0.246***	0.011	0.111***	0.036	Vertical mismatch		0.177***	0.015	0.206***	0.015
Horizontal mismatch	0.259***	0.010	0.155***	0.031	Horizontal mismatch		0.215***	0.016	0.219***	0.015
Low wage	0.125***	0.017	0.021	0.062	Low wage		0.097***	0.018	0.071***	0.020
Temporary contract	-0.004	0.010	0.027	0.038	Temporary contract		0.015	0.015	0.029**	0.014

Robust standard errors in parentheses

*** p<0.01

** p<0.05

* p<0.1

Source: University Graduate Job Placement Survey 2014 and 2019 (INE). Own calculations.

In relation to age, when the two extreme strata are compared, it is observed that the coefficients tend to be accentuated among the youngest graduates in all variables except for low wages and temporary employment, which show greater relationship among graduates aged 35 and over. These small differences could be due to the fact that, among older graduates, there may be a higher proportion of people who completed their education motivated by personal satisfaction and the desire to learn or understand the world they live in and who did not think as much about the potential influence of their studies on their professional career.

In panel B of the same table, results are organized by branch of knowledge. At first glance, results are quite similar to each other. Educational mismatch (horizontal and vertical) shows the greatest influence on dissatisfaction with the university experience in all branches, although the coefficient is accentuated in the field of Health Sciences. Unemployment and low wages also have a positive, substantial and statistically significant relationship in all branches, with the exception of Health Sciences, where the ATET of low wages is not significant. The results of inactivity differ to a greater extent by branch, since in two of them (Science and Arts & Humanities) the coefficients are not significant. Additionally, in the case of temporary employment, the results are similar and a significant small relationship is observed only for Engineering & Architecture.

Panel C first displays results by university type, face-to-face or distance. This division is of special interest since the profile of students at distance universities in Spain differs from that of students at face-to-face universities in that the former tend to be older students with previous higher education and such characteristics influence their motivation to study at university. In this case, the differences in results are remarkable since in all the investigated treatment variables, with the exception of temporary employment where coefficients are not significant, the ATET of face-to-face universities is larger. In fact, the only two variables with statistically significant coefficients in the distance learning graduates sample are those related to educational mismatch, even though coefficient size is practically halved compared to those estimated for face-to-face universities. These results suggest that the association of job placement results with satisfaction with university education tends to be greater among people who pursue higher education as an investment (professional future) than among those who consider it a consumer decision (personal satisfaction). The last analysis takes the duration of undergraduate studies (3 and 5 years) in the 2014 Bachelor’s sample into account. No remarkable differences are observed here, except in that in four of the studied variables coefficient size tends to be greater among graduates who completed longer degrees.

## Conclusions and final reflections

The Spanish university system has experienced extraordinary growth in recent decades, with the volume of enrolled students practically doubling between the 1985–86 (854,189 students) and 2020–21 (1,679,518 students) academic years [57]. In a context of “massification” and growing “marketization” of university systems, obtaining information on student satisfaction and researching its determinants may be essential for the long-term survival of higher education institutions. In this work, it has been shown that employability plays a very important role in graduate satisfaction with their university experience. Therefore, if we want to improve the satisfaction of our students, we should try to enhance the job placement processes of university graduates.

Among the six job placement indicators that have been studied, the two variables showing the greatest relationship with university experience dissatisfaction are vertical and horizontal educational mismatch. The fact that graduates are forced to work in lower-skilled jobs or outside the field of their studies significantly increases dissatisfaction with received education. Unemployment and earning low wages also affect satisfaction with the university experience negatively, although to a lesser extent. On the other hand, the sensitivity analysis that was carried out invites us to interpret the results for inactivity and temporary employment with more caution. The latter, in fact, has no influence on most graduates’ satisfaction.

In this article, the propensity score matching technique has been implemented in order to study the association between job placement outcomes and graduates’ dissatisfaction with their university education. In addition, a valuable source of information of extraordinary wealth and representative of the Spanish university system has been used. The results found are robust insofar as they are very stable regardless of the matching algorithms used. Furthermore, they have been observed in the three samples analyzed. In this sense, it should be emphasized that labor market transition results have a significant relationship with graduate satisfaction both during periods of employment growth (2014 to 2019) and periods of crisis (2010 to 2014).

The analysis of heterogeneity of coefficient sizes based on different socio-demographic and educational characteristics confirms that the graduates’ job placement outcomes have a significant influence on satisfaction regardless of sex, age, branch of knowledge and duration of studies. On the other hand, results change drastically depending on the type of university, with a noticeably greater relationship being found among graduates at face-to-face universities than at distance universities. In Spain, distance university students tend to be older and to have prior university studies. Therefore, the previous conclusion should be qualified by taking into account what motivated graduates when pursuing their education. Job placement outcomes determine satisfaction with the university experience among the students who consider higher education as an investment, and not among those who see it as a consumer decision. In this sense, it would be interesting to extend the research to other university systems in which the public sector plays a secondary role in financing higher education, especially in the Anglo-Saxon world, in order to assess whether there is a relationship between the economic benefits of the training received and satisfaction with the university experience.

This paper shows the importance of labor outcomes 4–5 years after graduation for university dissatisfaction. However, this association can certainly vary during graduates’ lives. For instance, going to university may have higher returns in the long term (10 or 20 years after completing their degree) than in the medium term (4 to 5 years after finishing their studies), the latter being this paper’s subject of research. One might wonder whether the sign and magnitude of estimates may vary depending on what stage of life is analyzed. In order to offer an adequate answer to this question, the analysis would have to be extended to a broader period of the graduates’ working lives, which we cannot address at this time given the limitations of available statistical information. However, we believe that, in principle, results would not essentially change for the reasons set out below.

First of all, the perspective from which we have approached this research is that of graduates who present unsatisfactory job placement results in terms of activity, employment, educational mismatch, wages and temporary employment. The empirical evidence available for the Spanish case indicates that 2 out of 3 young people start their working career in precarious jobs, a situation that tends to improve in the first five years of their working life. However, from the fifth year on, this positive trend fades and most young people who have not left the trap of precariousness, get stuck in such “bad jobs” long-term [58]. The EILU (INE) provides information on young graduates’ employment situation in their first job as well as 4–5 years after finishing their studies. The results of the survey in relation to the graduates of the 2013/14 academic year show a significant improvement in their employment situation between their first job and their current job, which is reflected in a 16-percentage-point reduction of vertical mismatch, a 9-point reduction of horizontal mismatch, a 15-point decrease in temporary contracts and, for the sake of mentioning one last indicator, an increase in nominal wages by more than 50%. Possibly, for most young people who still have jobs for which they are overqualified 5 years after finishing their studies, the problem is no longer temporary, and as their human capital depreciates, it will become permanent. The same reasoning could also be applied to graduates working outside their own field of studies or with unstable or poorly paid jobs in the medium term. For this reason, we tend to believe that most graduates who have precarious jobs 5 years after completing their studies will not be able to turn their precarious situation around in the long term. In this sense, the work trajectories of graduates who are successful in their job placement process will distance themselves from those of the previously mentioned graduates, which is why we anticipate that the sign and magnitude of the estimates obtained in this work will be maintained or even could increase in the long term.

In this work, the importance of employability as a determinant of the satisfaction of university graduates has been underlined. However, the foregoing should not be interpreted to mean that we should only pay attention to these types of factors. In fact, in some fields of study, such as Arts and Humanities, job placement results are usually not good, while graduates show above-average satisfaction with their university experience. It should be considered that expectations regarding job placement results tend to be lower among students in these fields of study, and also that having a job with other enjoyable attributes makes up for the potential negative association of job outcomes with satisfaction.

In this regard, the partial nature of the variables used to measure success in the job placement process must be mentioned as this work’s first limitation. Moreover, not having been able to analyze the influence of educational process variables on graduate satisfaction due to a lack of information should also be pointed out as a limitation. On the other hand, it would be advisable to be cautious in the interpretation of our results, insofar as the empirical approach has been based on a selection-on-observables methodology. As noted in the methodology section, in the event of a correlation between, on the one hand, satisfaction with university studies and job placement outcomes and, on the other hand, unobservable characteristics such as preferences, innate ability or other socio-demographic characteristics not taken into account in the analysis, results would be biased, especially when the dependable variable is a subjective indicator, which exacerbates the role of unobservables.

As for university policy recommendations, the strategic nature of employability should be emphasized in order to improve student satisfaction with their university experience. In this regard, academic authorities should gradually adapt their offer of studies to long-term changes in labor demand. Moreover, the dissemination of information on both the satisfaction of university students and the results of their transition into the labor market is especially necessary in countries where young people have difficulties developing satisfactory career paths. On the other hand, the institutional framework regulating the labor market should be improved, and the weaknesses of the Spanish productive sector, dominated by small companies which allocate few resources to R&D and which specialize in low value added activities, should be corrected. In short, a shift in demand for qualified work should be favored so that the growing supply of graduates from the Spanish university system can be absorbed. These proposals have a special meaning at a time of uncertainty such as the current one, when yet another economic crisis is hitting young people in Spain. According to data from the Spanish Labor Force Survey (INE), in the last two years, employment rates of young people aged 16 to 24 have experienced the largest relative drop as a result of the COVID-19 crisis, even though they had not yet fully recovered from the effects of the previous crisis. The economic authorities must act to minimize the impact of the crisis on the youth labor market and, in parallel, managers of university institutions must work to make the job placement results of our graduates more satisfactory in the medium term.

## Supporting information

S1 Annex (DOCX)
