# Supplementary material for: Graduate labour market outcomes and satisfaction with university education in Spain
Source: PLoS One. 2022 Jul 6;17(7):e0270643. doi: 10.1371/journal.pone.0270643 (PMC9258889; doi:10.1371/journal.pone.0270643)
Supplement: S1 Annex — (DOCX) [file pone.0270643.s001.docx]

**S1 Annex**

**Fig A1. Overlap between treatment and control distributions in estimating the association between inactivity and dissatisfaction with university education (Bachelor 2014)**

Source: University Graduate Job Placement Survey 2014 (INE). Own calculations.


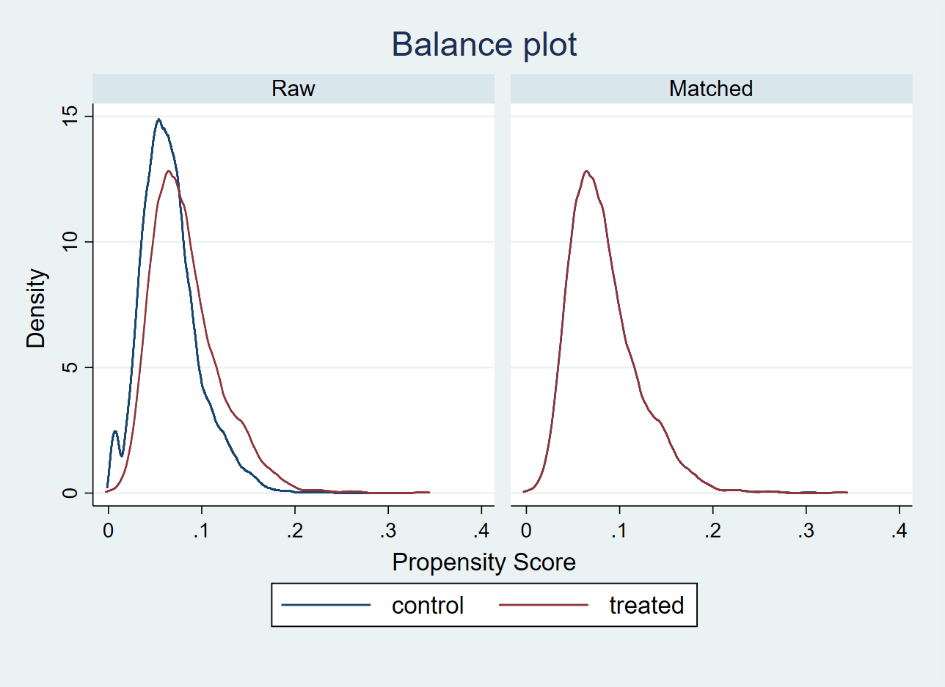


**Fig A2. Overlap between treatment and control distributions in estimating the association between unemployment and dissatisfaction with university education (Bachelor 2014)**

Source: University Graduate Job Placement Survey 2014 (INE). Own calculations.


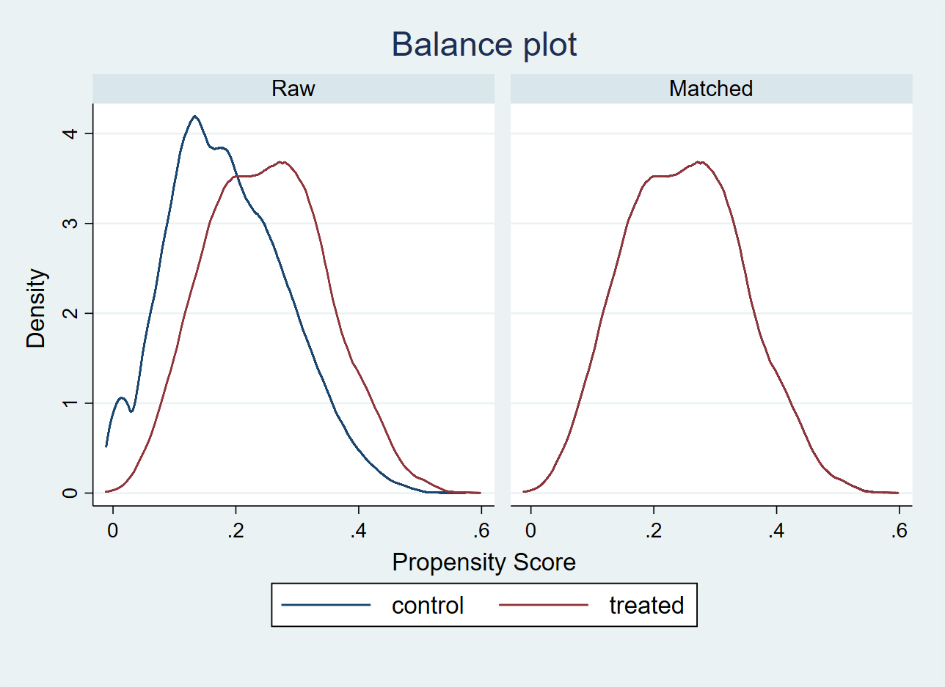


**Fig A3. Overlap between treatment and control distributions in estimating the association between vertical mismatch and dissatisfaction with university education (Bachelor 2014)**

Source: University Graduate Job Placement Survey 2014 (INE). Own calculations.


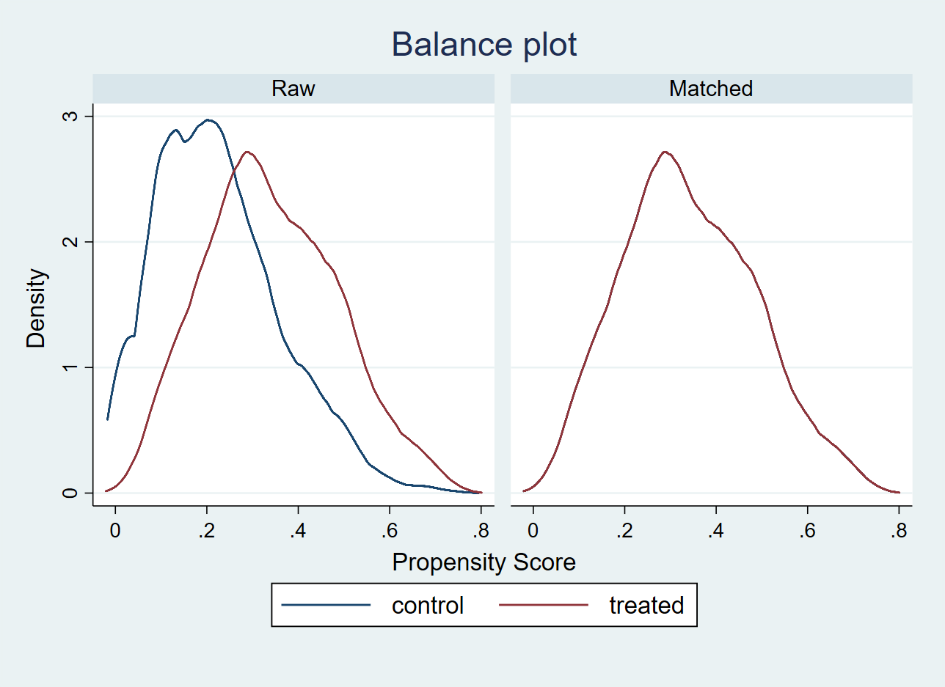


**Fig A4. Overlap between treatment and control distributions in estimating the association between horizontal mismatch and dissatisfaction with university education (Bachelor 2014)**

Source: University Graduate Job Placement Survey 2014 (INE). Own calculations.


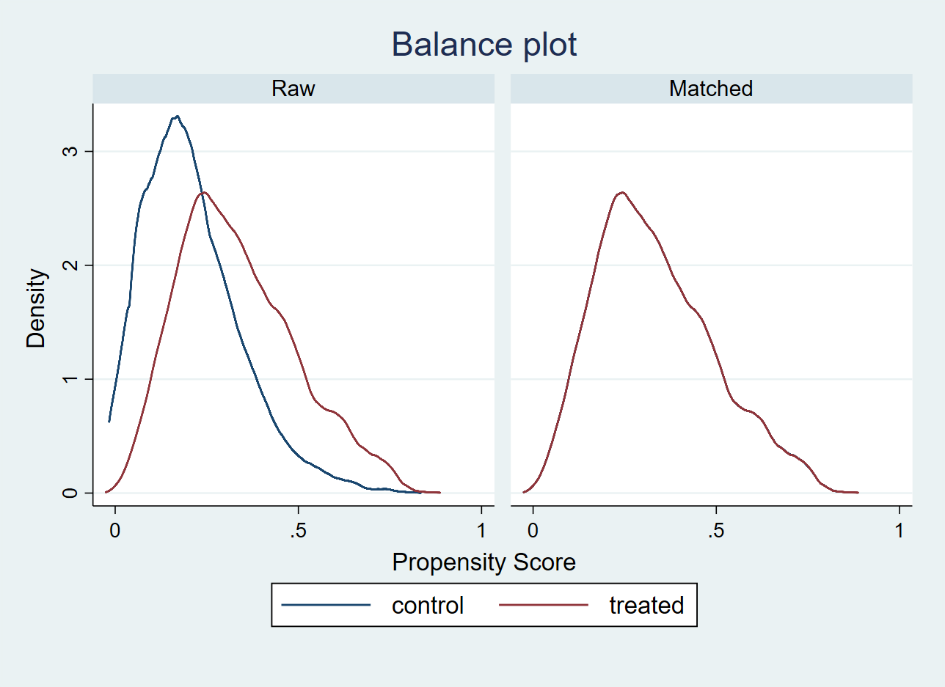


**Fig A5. Overlap between treatment and control distributions in estimating the association between earning a low wage and dissatisfaction with university education (Bachelor 2014)**

Source: University Graduate Job Placement Survey 2014 (INE). Own calculations.


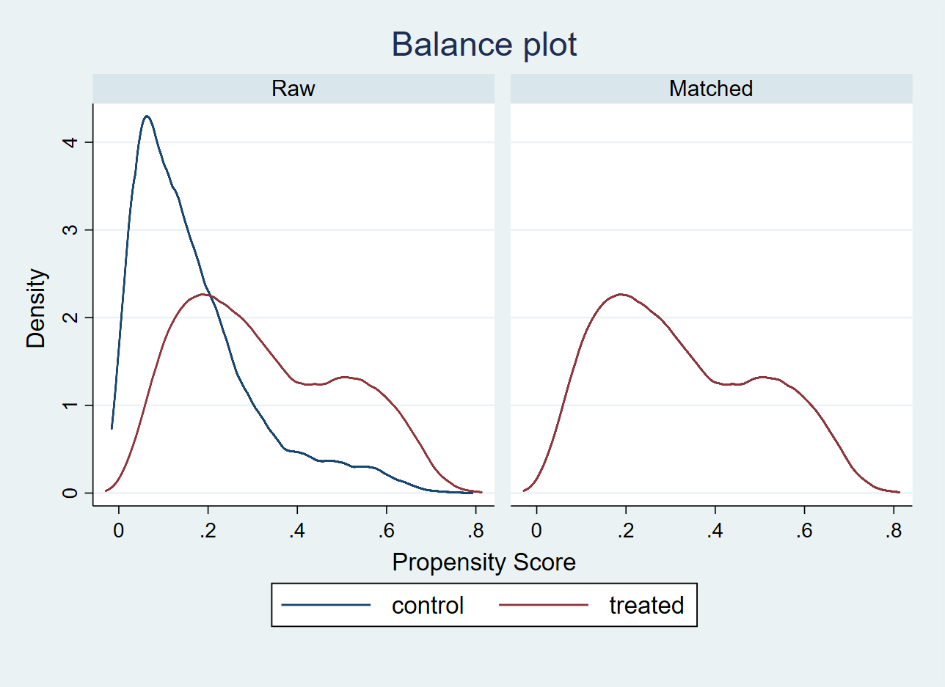


**Fig A6. Overlap between treatment and control distributions in estimating the association between having a temporary contract and dissatisfaction with university education (Bachelor 2014)**

Source: University Graduate Job Placement Survey 2014 (INE). Own calculations.


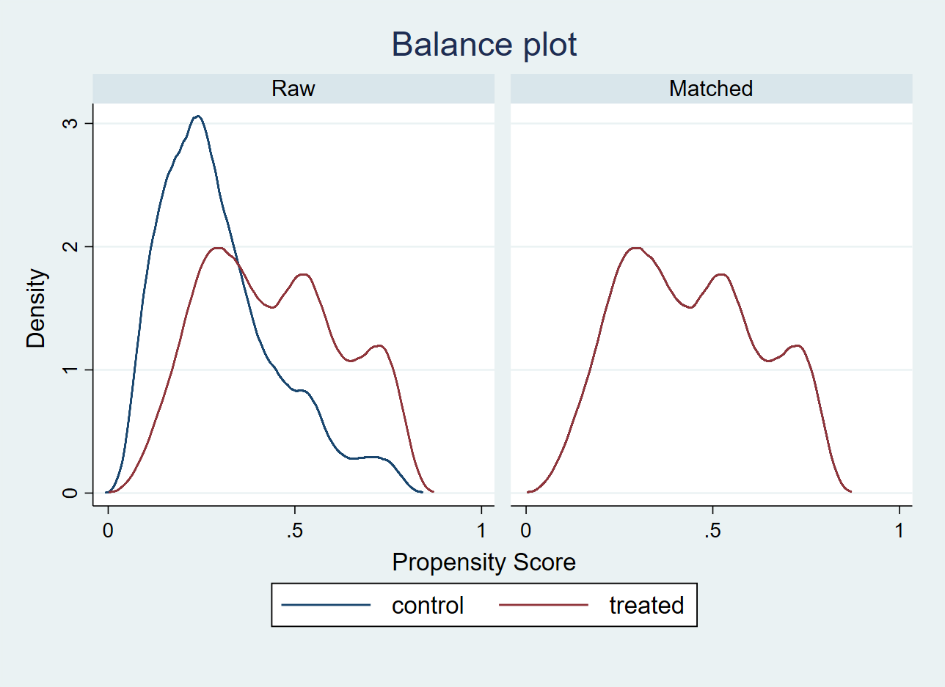


**Fig A7. Overlap between treatment and control distributions in estimating the association between inactivity and dissatisfaction with university education (Bachelor 2019)**

Source: University Graduate Job Placement Survey 2019 (INE). Own calculations.


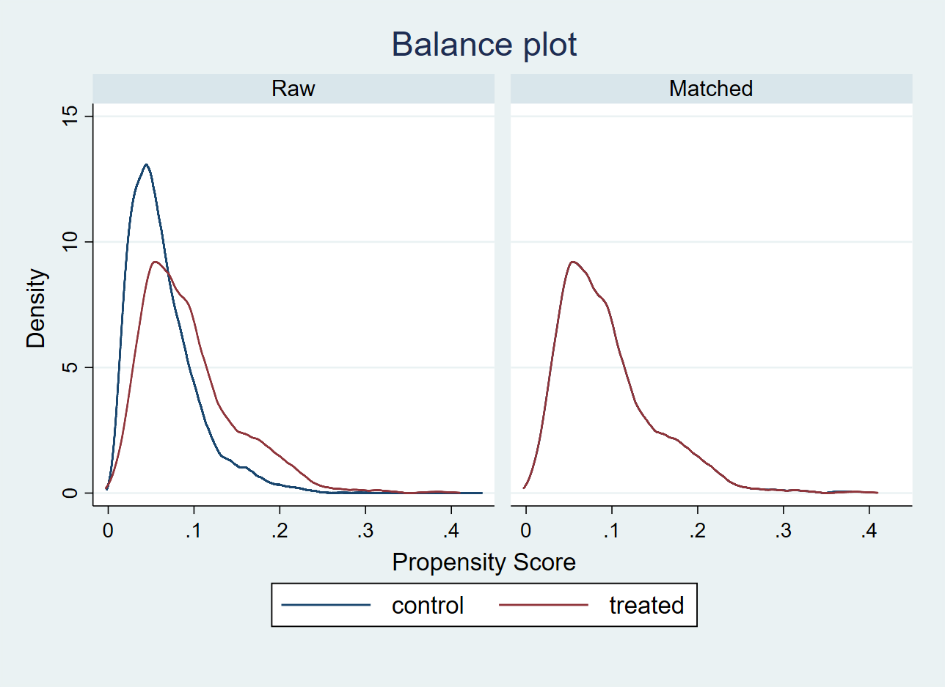


**Fig A8. Overlap between treatment and control distributions in estimating the association between unemployment and dissatisfaction with university education (Bachelor 2019)**

Source: University Graduate Job Placement Survey 2019 (INE). Own calculations.


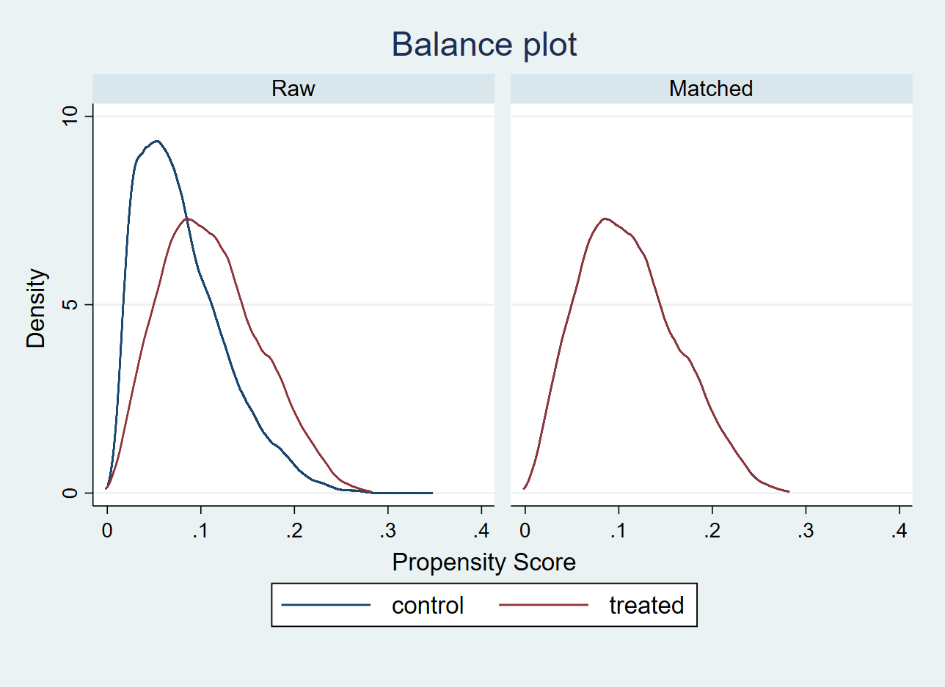


**Fig A9. Overlap between treatment and control distributions in estimating the association between vertical mismatch and dissatisfaction with university education (Bachelor 2019)**

Source: University Graduate Job Placement Survey 2019 (INE). Own calculations.


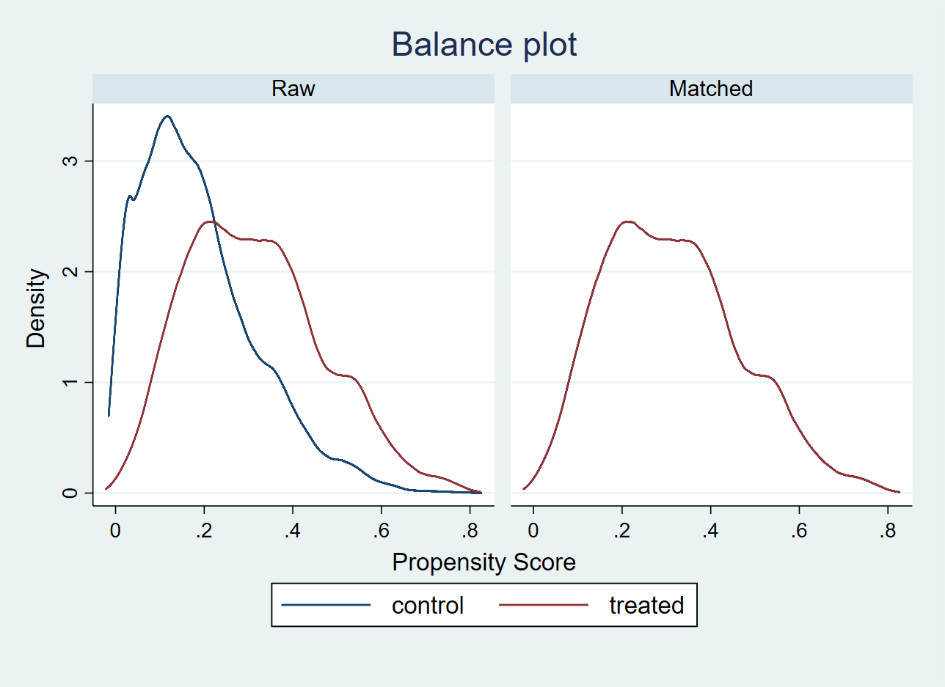


**Fig A10. Overlap between treatment and control distributions in estimating the association between horizontal mismatch and dissatisfaction with university education (Bachelor 2019)**

Source: University Graduate Job Placement Survey 2019 (INE). Own calculations.


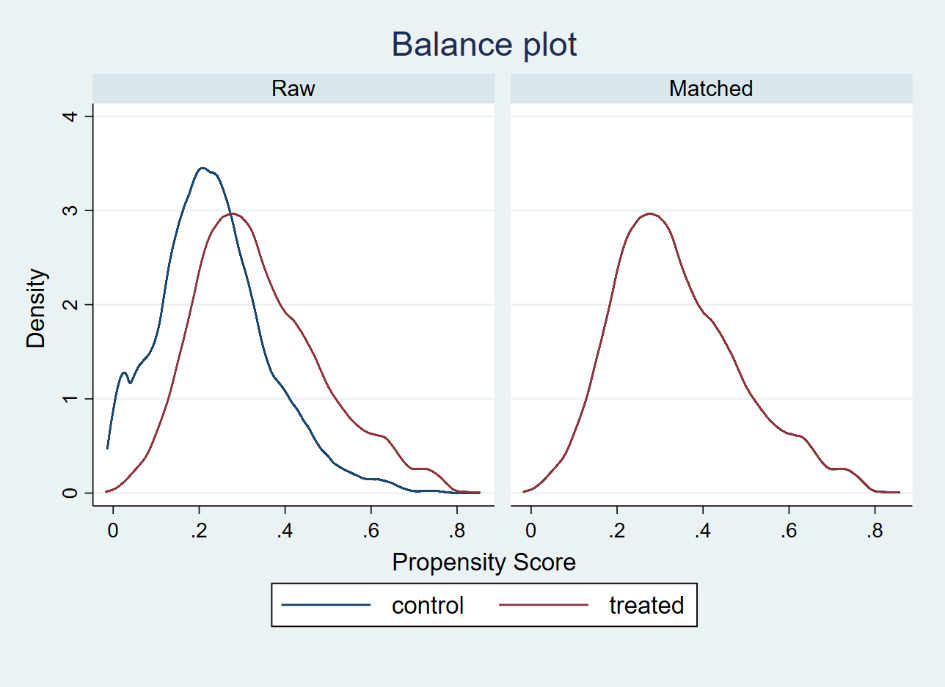


**Fig A11. Overlap between treatment and control distributions in estimating the association between earning a low wage and dissatisfaction with university education (Bachelor 2019)**

Source: University Graduate Job Placement Survey 2019 (INE). Own calculations.


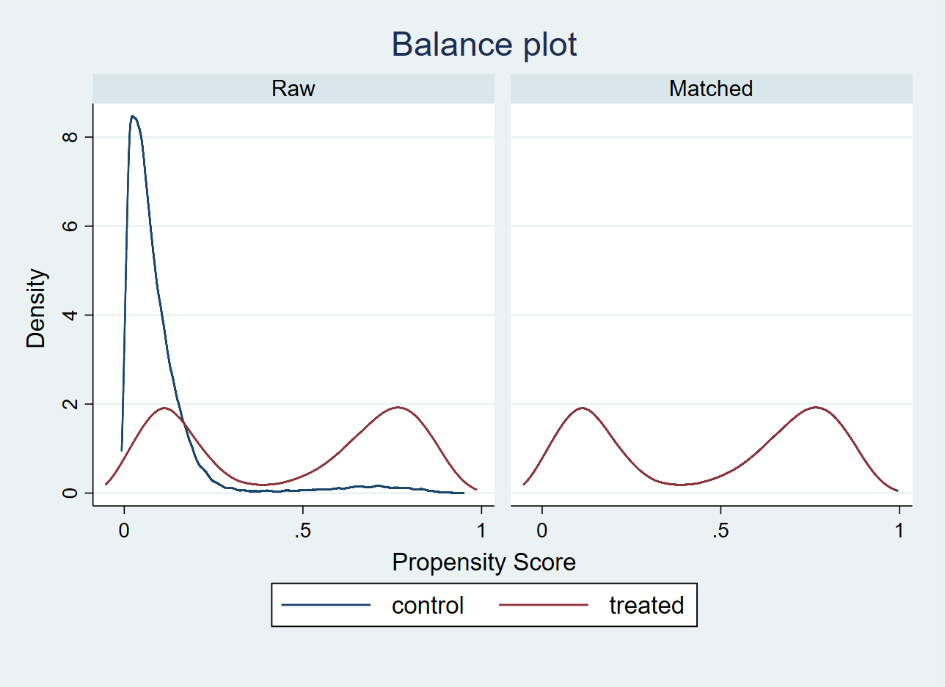


**Fig A12. Overlap between treatment and control distributions in estimating the association between having a temporary contract and dissatisfaction with university education (Bachelor 2019)**

Source: University Graduate Job Placement Survey 2019 (INE). Own calculations.


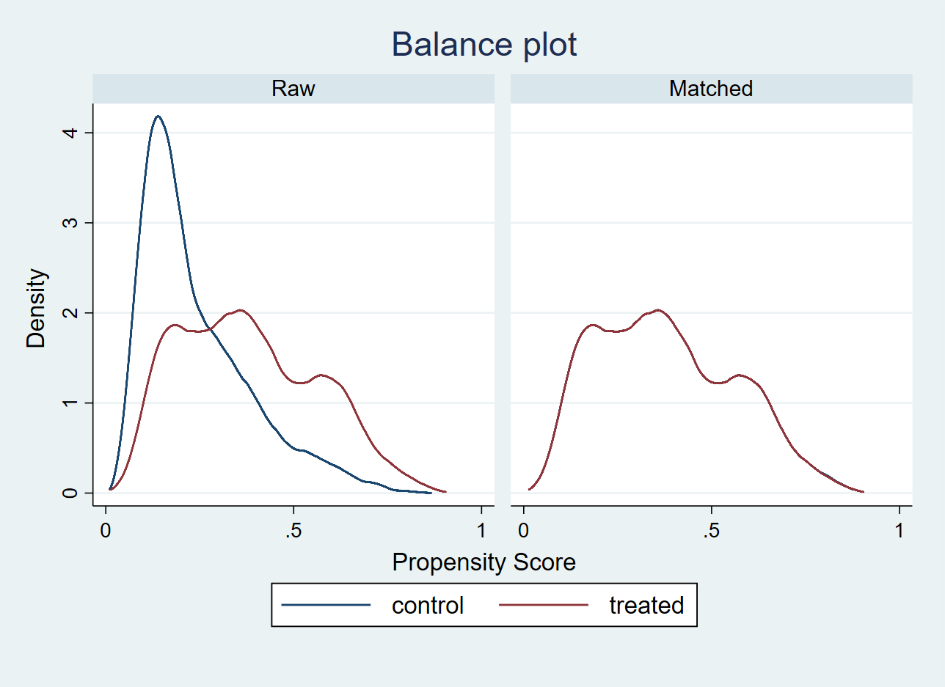


**Fig A13. Overlap between treatment and control distributions in estimating the association between inactivity and dissatisfaction with university education (Master 2019)**

Source: University Graduate Job Placement Survey 2019 (INE). Own calculations.


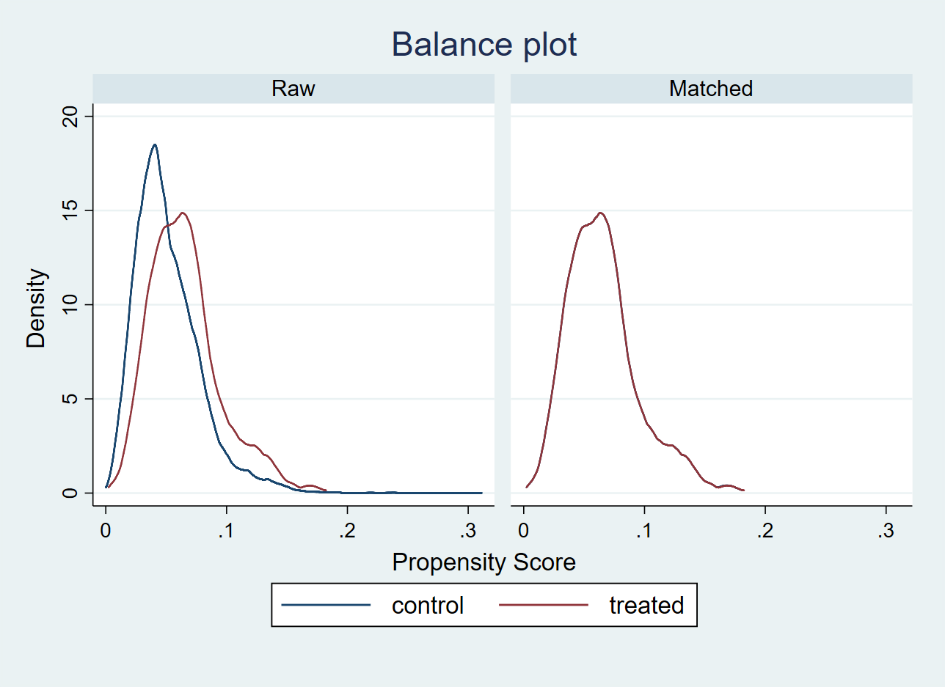


**Fig A14. Overlap between treatment and control distributions in estimating the association between unemployment and dissatisfaction with university education (Master 2019)**

Source: University Graduate Job Placement Survey 2019 (INE). Own calculations.


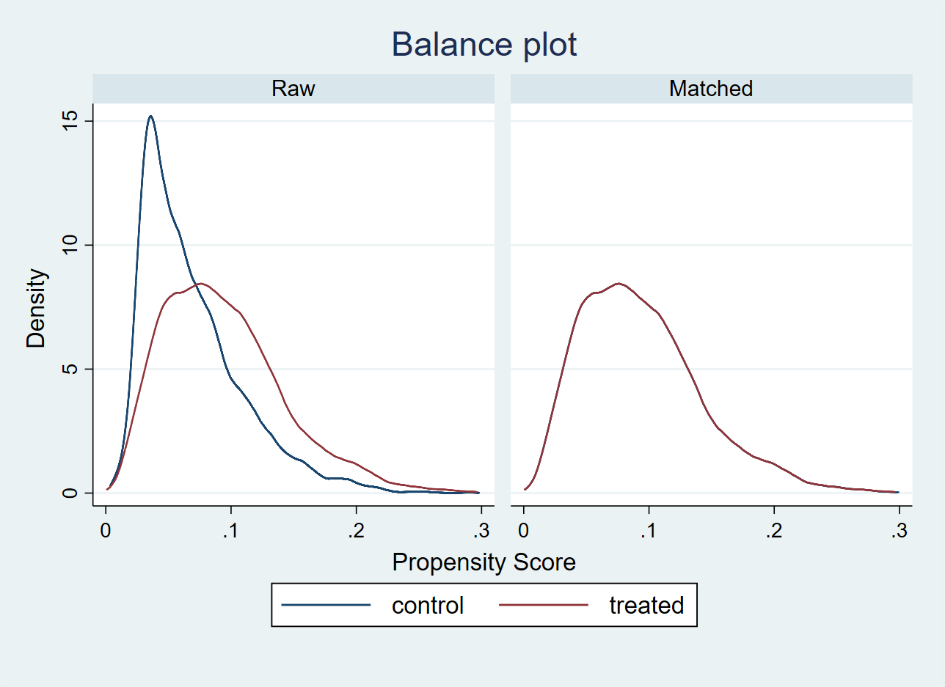


**Fig A15. Overlap between treatment and control distributions in estimating the association between vertical mismatch and dissatisfaction with university education (Master 2019)**

Source: University Graduate Job Placement Survey 2019 (INE). Own calculations.


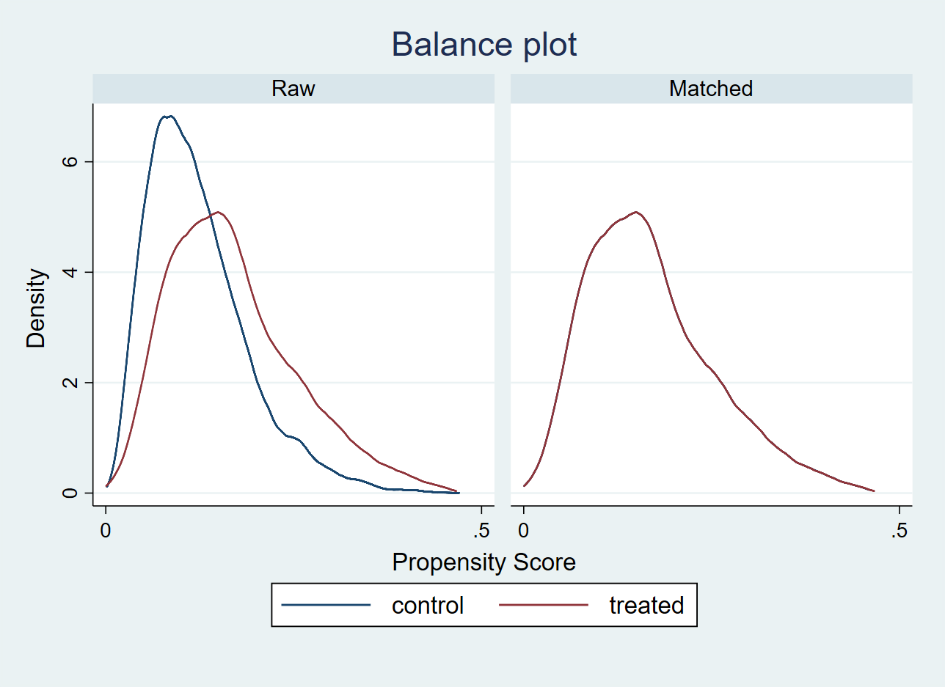


**Fig A16. Overlap between treatment and control distributions in estimating the association between horizontal mismatch and dissatisfaction with university education (Master 2019)**

Source: University Graduate Job Placement Survey 2019 (INE). Own calculations.


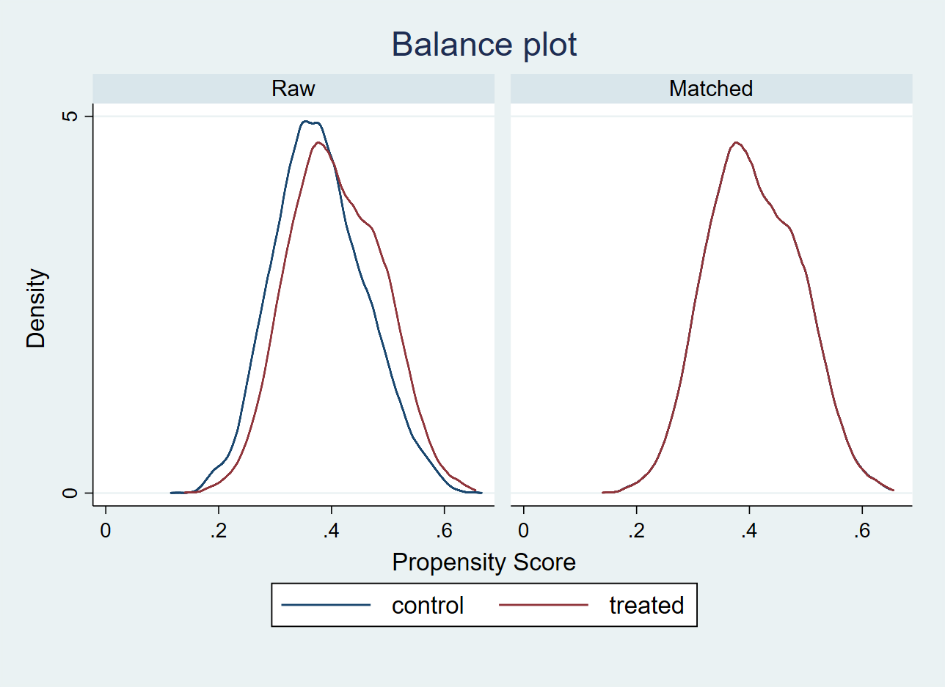


**Fig A17. Overlap between treatment and control distributions in estimating the association between earning a low wage and dissatisfaction with university education (Master 2019)**

Source: University Graduate Job Placement Survey 2019 (INE). Own calculations.


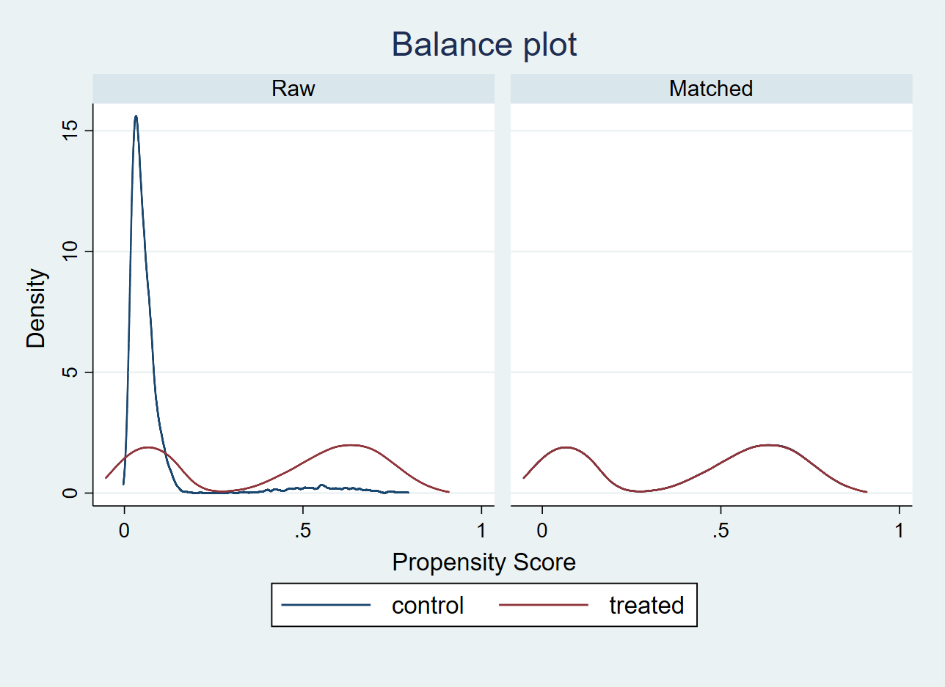


**Fig A18. Overlap between treatment and control distributions in estimating the association between having a temporary contract and dissatisfaction with university education (Master 2019)**

Source: University Graduate Job Placement Survey 2019 (INE). Own calculations.


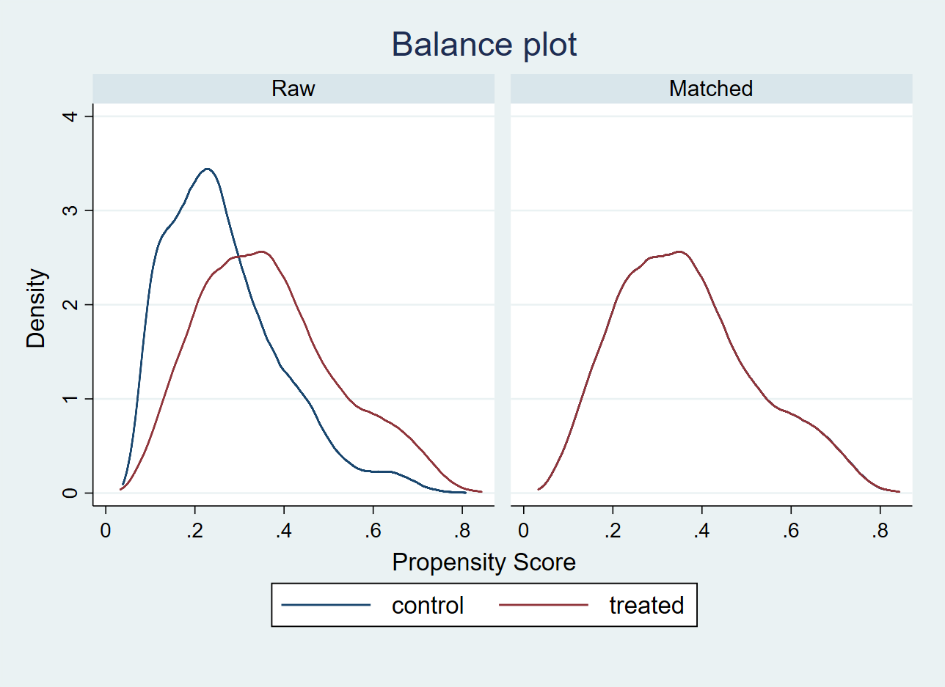


**A1. Marginal effects after probit (Master 2019)**

|  | (1) | (2) | (3) | (4) | (5) | (6) |
| --- | --- | --- | --- | --- | --- | --- |
| VARIABLES | INACTIVITY | UNEMPLOYMENT | VERTICAL MISM. | HORIZONTAL MISM. | LOW WAGE | TEMPORARY C. |
|  |  |  |  |  |  |  |
| Pr (y=1) | 0.047 | 0.063 | 0.113 | 0.388 | 0.068 | 0.276 |
|  |  |  |  |  |  |  |
| female | 0.007* | 0.003 | 0.002 | -0.012 | 0.008 | 0.014 |
| age 30 to 34 | -0.000 | -0.001 | 0.020*** | 0.030** | 0.001 | -0.051*** |
| age more than 34 | -0.007* | -0.009 | -0.008 | 0.047*** | -0.020** | -0.097*** |
| father higher edu | -0.000 | 0.001 | -0.029*** | -0.035** | -0.011* | -0.025 |
| mother higher edu | 0.007 | -0.012** | -0.034*** | -0.003 | -0.010 | 0.017 |
| father primary edu | -0.002 | 0.005 | 0.023*** | -0.000 | 0.005 | 0.009 |
| mother primary edu | 0.010*** | -0.011* | -0.002 | 0.008 | -0.007 | -0.002 |
| general grant | 0.005 | 0.017** | 0.011 | -0.014* | 0.013** | 0.039*** |
| excellence scholarship | 0.003 | 0.032* | -0.043* | -0.095*** | 0.004 | 0.043* |
| master | 0.002 | 0.002 | -0.041*** | 0.024 | -0.006 | 0.011 |
| private university | -0.010*** | -0.023*** | -0.030*** | -0.010 | -0.017** | -0.061*** |
| disable | 0.069*** | 0.046 | -0.024 | 0.000 | 0.039* | -0.029 |
| foreign | 0.020 | 0.026** | 0.023 | -0.030 | 0.022 | 0.002 |
| distance university | -0.007 | 0.007 | 0.027 | 0.071 | -0.013 | 0.021 |
| part-time job |  |  | 0.085*** | 0.079* | 0.516*** | 0.233*** |
| national university | -0.002 | -0.029*** | -0.047*** | -0.031 | -0.008 | -0.030 |
| Andalucía | 0.017 | 0.032*** | 0.017 | 0.064*** | 0.022 | 0.063*** |
| Aragón | -0.015 | -0.019 | 0.010 | 0.105** | -0.006 | 0.090* |
| Asturias | 0.010 | 0.018 | 0.013 | 0.105*** | 0.015 | 0.044 |
| Baleares | -0.011 | -0.026*** | -0.007 | 0.033 | -0.030 | 0.069 |
| Canarias | -0.018** | -0.017* | 0.002 | 0.105*** | 0.025 | 0.038 |
| Cantabria | 0.001 | 0.056** | 0.004 | 0.054 | -0.013 | 0.160*** |
| Castilla y León | 0.011 | -0.000 | 0.000 | 0.094*** | 0.024 | 0.040* |
| Castilla-La Mancha | 0.016 | -0.029** | 0.022 | 0.074 | 0.003 | 0.096** |
| Cataluña | -0.014 | -0.021* | -0.022 | -0.016 | -0.007 | 0.017 |
| C. Valenciana | -0.001 | 0.004 | 0.007 | 0.046 | 0.012 | 0.010 |
| Extremadura | -0.014 | -0.017 | 0.037 | 0.127* | 0.044* | 0.125*** |
| Galicia | 0.004 | -0.009 | 0.007 | 0.066*** | 0.019 | 0.039 |
| Murcia | 0.009 | 0.005 | -0.010 | 0.043** | 0.027 | 0.043 |
| Navarra | -0.017 | 0.009 | 0.018 | 0.091*** | 0.004 | 0.070** |
| País Vasco | 0.011 | -0.002 | 0.018 | 0.011 | -0.015 | 0.082** |
| Rioja (La) | -0.029** | -0.019 | -0.033 | -0.017 | -0.022* | 0.054 |
| Arts and Humanides | 0.040*** | 0.059*** | 0.083*** | 0.129*** | 0.056*** | -0.090*** |
| Social Sc., journalism, docum. | 0.001 | 0.046*** | 0.058*** | 0.136*** | 0.042*** | -0.104*** |
| Business, Admin., Law | 0.008*** | -0.006*** | 0.046*** | 0.036** | 0.042*** | -0.184*** |
| Sciences | 0.008*** | 0.059*** | 0.022*** | 0.054*** | 0.038*** | 0.020*** |
| Computer Science | -0.024*** | -0.032*** | -0.029*** | -0.089*** | -0.035*** | -0.154*** |
| Engineering, Ind. and Constr. | -0.021*** | -0.002 | 0.006 | 0.072*** | -0.002 | -0.108*** |
| Agriculture, livestock, fishing | -0.016*** | 0.006* | -0.004 | 0.006 | 0.017*** | -0.069*** |
| Health and Social Services | -0.009*** | 0.008*** | -0.042*** | 0.015** | -0.007*** | -0.048*** |
| Services | -0.014*** | -0.005** | 0.094*** | 0.152*** | 0.018*** | -0.125*** |
|  |  |  |  |  |  |  |
| Observations | 10,630 | 10,077 | 9,307 | 9,289 | 9,119 | 9,363 |
| Pseudo R^2^ | 0.0346 | 0.0459 | 0.0572 | 0.0212 | 0.3142 | 0.0822 |

Robust standard errors in parentheses

*** p<0.01, ** p<0.05, * p<0.1

Source: University Graduate Job Placement Survey 2019 (INE). Own calculations.

**A2. Standardized mean differences before and after matching (Master 2019)**

| *Covariate balance summary* | | | | | | | | | | | | | | | | | | |
| --- | --- | --- | --- | --- | --- | --- | --- | --- | --- | --- | --- | --- | --- | --- | --- | --- | --- | --- |
|  | INACTIVITY | |  | UNEMPLOYMENT | | |  | VERTICAL MISM. | |  | HORIZONTAL MISM. | |  | LOW WAGE | |  | TEMPORARY C. | |
|  | Raw | Matched |  | Raw | Matched | |  | Raw | Matched |  | Raw | Matched |  | Raw | Matched |  | Raw | Matched |
| Number of obs. | 10,630 | 1,106 |  | 10,077 | 1,428 | |  | 9,307 | 2,390 |  | 9,289 | 7,260 |  | 9,119 | 2,208 |  | 9,363 | 5,530 |
| Treated obs. | 553 | 553 |  | 714 | 714 | |  | 1,195 | 1,195 |  | 3,630 | 3,630 |  | 1,104 | 1,104 |  | 2,765 | 2,765 |
| Control obs. | 10,077 | 553 |  | 9,363 | 714 | |  | 8,112 | 1,195 |  | 5,659 | 3,630 |  | 8,015 | 1,104 |  | 6,598 | 2,765 |
| *Standardized differences* | | | | | | | | | | | | | | | | | | |
|  | INACTIVITY | |  | UNEMPLOYMENT | | |  | VERTICAL MISM. | |  | HORIZONTAL MISM. | |  | LOW WAGE | |  | TEMPORARY C. | |
| covariates | Raw | Matched |  | Raw | | Matched |  | Raw | Matched |  | Raw | Matched |  | Raw | Matched |  | Raw | Matched |
| mother primary edu | 0.046 | 0.074 |  | -0.05 | | 0.06 |  | 0.14 | 0.00 |  | 0.07 | -0.02 |  | -0.04 | 0.01 |  | -0.05 | 0.00 |
| mother higher edu | 0.041 | -0.141 |  | -0.07 | | -0.01 |  | -0.26 | -0.01 |  | -0.06 | 0.02 |  | -0.10 | -0.02 |  | 0.00 | 0.03 |
| father primary edu | 0.022 | 0.084 |  | 0.02 | | 0.05 |  | 0.19 | 0.00 |  | 0.06 | -0.04 |  | 0.01 | 0.07 |  | 0.00 | 0.00 |
| father higher edu | 0.013 | -0.121 |  | -0.04 | | 0.02 |  | -0.27 | 0.00 |  | -0.09 | 0.04 |  | -0.11 | -0.06 |  | -0.07 | 0.02 |
| disability | 0.099 | -0.024 |  | 0.05 | | -0.03 |  | -0.03 | -0.01 |  | 0.01 | 0.00 |  | 0.02 | 0.05 |  | -0.03 | 0.00 |
| female | 0.095 | -0.088 |  | 0.06 | | -0.04 |  | 0.04 | 0.01 |  | -0.02 | -0.01 |  | 0.22 | -0.11 |  | 0.13 | -0.01 |
| age 30 to 34 | 0.042 | -0.080 |  | 0.05 | | -0.06 |  | 0.11 | -0.02 |  | -0.01 | 0.01 |  | 0.09 | 0.09 |  | 0.03 | -0.03 |
| age more than 34 | -0.121 | 0.108 |  | -0.16 | | -0.02 |  | -0.10 | -0.01 |  | 0.08 | -0.04 |  | -0.25 | -0.09 |  | -0.27 | 0.00 |
| foreign | 0.075 | -0.009 |  | 0.07 | | 0.05 |  | 0.01 | 0.04 |  | -0.02 | 0.02 |  | 0.06 | -0.03 |  | -0.01 | 0.04 |
| general grant | 0.106 | 0.041 |  | 0.22 | | -0.02 |  | 0.19 | -0.02 |  | -0.01 | 0.03 |  | 0.27 | -0.04 |  | 0.20 | 0.00 |
| excellence scholarship | 0.024 | 0.078 |  | 0.10 | | 0.06 |  | -0.07 | 0.00 |  | -0.07 | 0.02 |  | 0.00 | 0.02 |  | 0.04 | 0.06 |
| private university | -0.204 | -0.033 |  | -0.29 | | 0.02 |  | -0.16 | -0.03 |  | -0.03 | -0.02 |  | -0.26 | -0.06 |  | -0.25 | 0.01 |
| distance university | -0.158 | -0.007 |  | -0.19 | | 0.00 |  | -0.09 | -0.02 |  | 0.03 | 0.00 |  | -0.23 | 0.00 |  | -0.14 | 0.02 |
| master | 0.033 | 0.027 |  | 0.00 | | -0.01 |  | -0.21 | -0.01 |  | 0.05 | 0.03 |  | -0.01 | 0.00 |  | 0.02 | 0.01 |
| part-time job | - | - |  | - | | - |  | 0.27 | -0.02 |  | 0.11 | -0.02 |  | 1.50 | 0.00 |  | 0.42 | 0.03 |
| Arts and Humanides | 0.275 | 0.072 |  | 0.19 | | -0.03 |  | 0.16 | -0.06 |  | 0.10 | -0.02 |  | 0.23 | -0.03 |  | 0.01 | 0.01 |
| Social Sc., journalism, docum. | 0.004 | -0.006 |  | 0.13 | | 0.03 |  | 0.11 | 0.04 |  | 0.11 | 0.02 |  | 0.16 | -0.07 |  | -0.03 | -0.01 |
| Business, Admin., Law | 0.043 | -0.056 |  | -0.16 | | -0.01 |  | 0.00 | 0.01 |  | -0.05 | 0.02 |  | -0.12 | 0.00 |  | -0.31 | 0.05 |
| Sciences | 0.079 | 0.000 |  | 0.23 | | 0.04 |  | 0.00 | 0.00 |  | -0.02 | 0.00 |  | 0.01 | 0.02 |  | 0.20 | 0.00 |
| Computer Science | -0.126 | 0.016 |  | -0.15 | | 0.03 |  | -0.10 | -0.02 |  | -0.11 | -0.02 |  | -0.20 | 0.03 |  | -0.13 | 0.02 |
| Engineering, Ind. and Constr. | -0.178 | -0.028 |  | -0.09 | | 0.03 |  | -0.06 | 0.04 |  | 0.02 | 0.00 |  | -0.20 | 0.06 |  | -0.10 | -0.03 |
| Agriculture, livestock, fishing | -0.045 | 0.013 |  | 0.00 | | 0.06 |  | -0.02 | 0.00 |  | -0.03 | 0.00 |  | 0.01 | -0.01 |  | 0.02 | -0.01 |
| Health and Social Services | -0.069 | 0.032 |  | -0.04 | | -0.08 |  | -0.22 | 0.03 |  | -0.06 | 0.03 |  | -0.10 | 0.01 |  | 0.05 | -0.02 |
| Services | -0.114 | -0.008 |  | -0.11 | | -0.01 |  | 0.15 | 0.05 |  | 0.12 | 0.01 |  | -0.07 | 0.05 |  | -0.14 | 0.03 |
| National university | 0.000 | 0.000 |  | -0.08 | | -0.01 |  | -0.06 | 0.00 |  | 0.01 | -0.01 |  | -0.12 | 0.08 |  | -0.11 | 0.00 |
| Andalucía | 0.159 | -0.043 |  | 0.23 | | -0.05 |  | 0.09 | -0.07 |  | 0.02 | 0.00 |  | 0.09 | -0.04 |  | 0.09 | -0.05 |
| Aragón | -0.056 | -0.013 |  | -0.03 | | 0.03 |  | 0.02 | 0.01 |  | 0.04 | 0.01 |  | 0.02 | 0.04 |  | 0.07 | -0.02 |
| Asturias | 0.035 | 0.022 |  | 0.06 | | -0.01 |  | 0.02 | -0.03 |  | 0.03 | 0.03 |  | 0.02 | 0.04 |  | 0.02 | 0.00 |
| Baleares | -0.023 | 0.078 |  | -0.06 | | -0.03 |  | -0.01 | 0.07 |  | -0.01 | 0.00 |  | -0.10 | -0.07 |  | 0.03 | 0.00 |
| Canarias | -0.070 | 0.032 |  | -0.02 | | -0.04 |  | 0.01 | -0.02 |  | 0.04 | 0.04 |  | 0.07 | -0.03 |  | 0.03 | -0.02 |
| Cantabria | 0.027 | 0.000 |  | 0.11 | | 0.03 |  | 0.01 | -0.01 |  | 0.00 | 0.02 |  | 0.01 | 0.00 |  | 0.08 | 0.01 |
| Castilla y León | 0.050 | 0.013 |  | 0.00 | | -0.03 |  | 0.00 | 0.02 |  | 0.05 | -0.02 |  | 0.06 | 0.00 |  | 0.01 | 0.01 |
| Castilla-La Mancha | 0.074 | 0.020 |  | -0.07 | | -0.01 |  | 0.05 | 0.03 |  | 0.02 | 0.00 |  | 0.02 | 0.06 |  | 0.06 | -0.02 |
| Cataluña | -0.119 | 0.006 |  | -0.12 | | 0.00 |  | -0.11 | -0.01 |  | -0.09 | -0.01 |  | -0.07 | 0.03 |  | -0.03 | 0.00 |
| C. Valenciana | -0.005 | 0.094 |  | 0.03 | | 0.02 |  | 0.02 | 0.05 |  | 0.00 | 0.02 |  | 0.02 | 0.02 |  | -0.03 | 0.02 |
| Extremadura | -0.048 | -0.054 |  | -0.01 | | 0.06 |  | 0.07 | -0.01 |  | 0.06 | -0.03 |  | 0.13 | -0.03 |  | 0.10 | -0.01 |
| Galicia | 0.043 | -0.035 |  | 0.00 | | 0.02 |  | 0.07 | -0.03 |  | 0.04 | -0.03 |  | 0.11 | 0.06 |  | 0.03 | 0.04 |
| Murcia | 0.053 | 0.024 |  | 0.02 | | 0.01 |  | -0.02 | 0.02 |  | 0.01 | 0.02 |  | 0.08 | 0.00 |  | 0.01 | 0.00 |
| Navarra | -0.080 | -0.078 |  | 0.00 | | -0.01 |  | -0.02 | -0.02 |  | 0.02 | 0.03 |  | -0.06 | -0.01 |  | -0.01 | -0.04 |
| País Vasco | 0.042 | 0.040 |  | 0.00 | | 0.03 |  | 0.02 | -0.02 |  | -0.03 | 0.02 |  | -0.02 | -0.01 |  | 0.04 | 0.05 |
| Rioja (La) | -0.196 | 0.018 |  | -0.13 | | 0.01 |  | -0.08 | -0.03 |  | -0.01 | 0.01 |  | -0.13 | -0.08 |  | -0.03 | 0.00 |

Source: University Graduate Job Placement Survey 2019 (INE). Own calculations.

**A3. Marginal effects after probit (Bachelor 2014)**

|  | (1) | (2) | (3) | (4) | (5) | (6) |
| --- | --- | --- | --- | --- | --- | --- |
| VARIABLES | INACTIVITY | UNEMPLOYMENT | VERTICAL MISM. | HORIZONTAL MISM. | LOW WAGE | TEMPORARY C. |
|  |  |  |  |  |  |  |
| Pr (y=1) | 0.061 | 0.178 | 0.221 | 0.203 | 0.155 | 0.330 |
|  |  |  |  |  |  |  |
| female | 0.003 | 0.012 | 0.013* | 0.007 | 0.019*** | 0.041*** |
| age 30 to 34 | -0.018*** | 0.014* | 0.044*** | 0.038*** | -0.039*** | 0.001 |
| age more than 34 | -0.009** | -0.030** | 0.046*** | 0.075*** | -0.103*** | -0.135*** |
| general grant | -0.011*** | 0.041*** | 0.060*** | 0.018* | 0.035*** | 0.034*** |
| excellence scholarship | 0.004 | -0.060*** | -0.072*** | -0.038 | -0.030 | -0.055*** |
| master | -0.010* | 0.019*** | -0.066*** | -0.044*** | 0.033*** | 0.031*** |
| private university | -0.002 | -0.003 | -0.046** | -0.024* | 0.015 | -0.029*** |
| disability | 0.070*** | 0.073** | 0.050* | 0.023 | 0.022 | -0.001 |
| foreign | 0.023 | 0.048 | -0.078** | -0.039 | -0.010 | -0.098** |
| 4-year degree | 0.025** | 0.022 | -0.014 | -0.071*** | 0.033* | 0.029 |
| 3-year degree | 0.015*** | 0.096*** | 0.123*** | 0.103*** | 0.097*** | 0.052*** |
| part-time job | - | - | 0.136*** | 0.087*** | 0.223*** | 0.225*** |
| Andalucía | 0.017** | 0.124*** | 0.049*** | 0.006 | 0.097*** | 0.086*** |
| Aragón | 0.020* | -0.006 | 0.025 | -0.018 | -0.002 | 0.068*** |
| Asturias (Princ. de) | 0.024** | 0.088*** | 0.025 | 0.000 | 0.066** | 0.067*** |
| Balears (Illes) | 0.016 | -0.009 | 0.022 | -0.051*** | -0.029* | 0.057* |
| Canarias | 0.052*** | 0.108*** | 0.069** | 0.037* | 0.122*** | 0.050* |
| Cantabria | 0.007 | 0.072** | 0.023 | 0.004 | 0.130*** | 0.097*** |
| Castilla y León | 0.015 | 0.069*** | 0.022 | -0.001 | 0.069*** | 0.057*** |
| Castilla-La Mancha | 0.004 | 0.111*** | 0.052* | 0.046** | 0.048** | 0.108*** |
| Cataluña | 0.012* | -0.048*** | -0.005 | -0.035*** | -0.008 | 0.027** |
| Comunitat Valenciana | 0.021*** | 0.060*** | 0.037** | -0.016 | 0.065*** | 0.063*** |
| Extremadura | 0.012 | 0.116*** | 0.062** | 0.042* | 0.106*** | 0.097*** |
| Galicia | 0.016 | 0.082*** | 0.046** | -0.001 | 0.071*** | 0.068*** |
| Murcia (Región de) | 0.030*** | 0.078*** | 0.048** | 0.025 | 0.078*** | 0.069*** |
| Navarra (Com. Foral) | 0.027** | 0.009 | 0.017 | -0.036 | 0.043 | 0.099*** |
| País Vasco | 0.012* | 0.032*** | 0.023 | 0.001 | 0.060*** | 0.141*** |
| Rioja (La) | 0.024 | -0.027 | 0.033 | -0.004 | 0.006 | 0.082** |
| Distance universities | 0.024* | -0.008 | 0.017 | 0.122*** | -0.010 | 0.009 |
| Early education training | -0.043*** | -0.057*** | -0.039* | -0.087*** | -0.012 | 0.345*** |
| Primary edu. teacher training | -0.046*** | -0.052*** | -0.066*** | -0.042** | -0.024 | 0.327*** |
| Other training of teaching staff | -0.041*** | -0.061*** | -0.057*** | -0.044*** | -0.048*** | 0.334*** |
| Arts | -0.014*** | 0.133*** | 0.202*** | 0.206*** | 0.173*** | 0.169*** |
| Audiovisual techniques & media | -0.029*** | 0.105*** | 0.201*** | 0.218*** | 0.078*** | 0.205*** |
| Humanities | -0.005*** | 0.155*** | 0.200*** | 0.394*** | 0.071*** | 0.241*** |
| Languages | -0.027*** | 0.034*** | 0.001 | 0.098*** | 0.078*** | 0.282*** |
| Psychology | -0.043*** | 0.055*** | 0.071*** | 0.100*** | 0.077*** | 0.138*** |
| Economics | -0.031*** | -0.003 | 0.124*** | 0.032*** | -0.017*** | 0.138*** |
| Other social & behavioral sc. | -0.015*** | 0.045*** | 0.095*** | 0.344*** | 0.052*** | 0.208*** |
| Journalism | -0.046*** | 0.073*** | 0.063*** | 0.199*** | 0.059*** | 0.231*** |
| Business administration & manag. | -0.052*** | -0.042*** | 0.090*** | 0.022 | -0.061*** | 0.090*** |
| Other business education | -0.049*** | 0.008*** | 0.142*** | 0.242*** | 0.023*** | 0.146*** |
| Life sciences | -0.030*** | 0.109*** | 0.094*** | 0.125*** | 0.020*** | 0.229*** |
| Physical, chemical, geolog. sc. | -0.020*** | 0.045*** | 0.029*** | 0.052*** | -0.043*** | 0.214*** |
| Mathematics and statistics | -0.036*** | -0.049*** | -0.048*** | 0.014** | -0.036*** | 0.111*** |
| Computer Science | -0.052*** | -0.138*** | -0.079*** | -0.146*** | -0.131*** | 0.058*** |
| Engineering and related profess. | -0.055*** | -0.079*** | -0.084*** | -0.069*** | -0.107*** | 0.155*** |
| Manufacturing ind. & production | -0.057*** | -0.002 | 0.024* | 0.074*** | -0.064*** | 0.179*** |
| Architecture & construction | -0.041*** | -0.019*** | -0.029** | 0.014 | -0.056*** | 0.114*** |
| Agriculture, Livestock and fishing | -0.044*** | -0.011** | -0.020 | 0.033** | -0.043*** | 0.219*** |
| Veterinary | -0.043*** | -0.056*** | -0.145*** | -0.132*** | 0.025*** | 0.061*** |
| Medicine | -0.064*** | -0.184*** | -0.233*** | -0.217*** | -0.167*** | -0.081*** |
| Nursing and care for the sick | -0.042*** | -0.068*** | -0.188*** | -0.175*** | -0.104*** | 0.337*** |
| Other health sciences | -0.044*** | -0.104*** | -0.173*** | -0.130*** | -0.074*** | 0.030*** |
| Social work and counseling | -0.045*** | -0.007 | 0.094*** | 0.118*** | -0.030** | 0.256*** |
| Tourism and hospitality | -0.033*** | -0.035*** | 0.150*** | 0.109*** | -0.036*** | 0.221*** |
| Sports | -0.039*** | -0.051*** | 0.114*** | 0.032*** | 0.057*** | 0.292*** |
| Transport services | -0.031*** | 0.006 | -0.095*** | -0.110*** | -0.061*** | 0.244*** |
|  |  |  |  |  |  |  |
| Observations | 30,379 | 28,331 | 21,540 | 21,426 | 17,777 | 22,214 |
| Pseudo R^2^ | 0.032 | 0.067 | 0.107 | 0.116 | 0.156 | 0.115 |

Robust standard errors in parentheses

*** p<0.01, ** p<0.05, * p<0.1

Source: University Graduate Job Placement Survey 2014 (INE). Own calculations.

**A4. Standardized mean differences before and after matching (Bachelor 2014)**

| *Covariate balance summary* | | | | | | | | | | | | | | | | | |
| --- | --- | --- | --- | --- | --- | --- | --- | --- | --- | --- | --- | --- | --- | --- | --- | --- | --- |
|  | INACTIVITY | |  | UNEMPLOYMENT | |  | VERTICAL MISM. | |  | HORIZONTAL MISM. | |  | LOW WAGE | |  | TEMPORARY C. | |
|  | Raw | Matched |  | Raw | Matched |  | Raw | Matched |  | Raw | Matched |  | Raw | Matched |  | Raw | Matched |
| Number of obs | 30,379 | 4,096 |  | 28,331 | 11,304 |  | 21,540 | 10,954 |  | 21,426 | 10,234 |  | 17,777 | 7,080 |  | 22,214 | 15,446 |
| Treated obs | 2,048 | 2,048 |  | 5,652 | 5,652 |  | 5,477 | 5,477 |  | 5,117 | 5,117 |  | 3,540 | 3,540 |  | 7,723 | 7,723 |
| Control obs | 28,331 | 2,048 |  | 22,679 | 5,652 |  | 16,063 | 5,477 |  | 16,309 | 5,117 |  | 14,237 | 3,540 |  | 14,491 | 7,723 |
| *Standardized differences* | | | | | | | | | | | | | | | | | |
|  | INACTIVITY | |  | UNEMPLOYMENT | |  | VERTICAL MISM. | |  | HORIZONTAL MISM. | |  | LOW WAGE | |  | TEMPORARY C. | |
| covariates | Raw | Matched |  | Raw | Matched |  | Raw | Matched |  | Raw | Matched |  | Raw | Matched |  | Raw | Matched |
| female | 0.051 | 0.001 |  | 0.09 | -0.01 |  | 0.08 | -0.01 |  | 0.05 | -0.02 |  | 0.27 | -0.01 |  | 0.24 | -0.02 |
| age 30 to 34 | -0.132 | 0.001 |  | 0.05 | 0.03 |  | 0.07 | 0.01 |  | 0.03 | 0.06 |  | -0.12 | 0.09 |  | 0.00 | 0.00 |
| age more than 34 | 0.058 | 0.026 |  | -0.11 | 0.04 |  | 0.04 | -0.01 |  | 0.21 | 0.00 |  | -0.31 | 0.04 |  | -0.28 | 0.07 |
| general grant | -0.065 | 0.015 |  | 0.24 | 0.01 |  | 0.20 | 0.00 |  | 0.05 | 0.04 |  | 0.28 | 0.02 |  | 0.21 | 0.03 |
| excellence scholarship | -0.008 | 0.064 |  | -0.08 | 0.06 |  | -0.12 | 0.05 |  | -0.09 | 0.05 |  | -0.06 | 0.04 |  | -0.07 | 0.03 |
| master | -0.024 | 0.005 |  | 0.12 | 0.02 |  | -0.15 | 0.01 |  | -0.06 | 0.01 |  | 0.16 | 0.00 |  | 0.08 | 0.03 |
| private university | -0.021 | 0.044 |  | -0.15 | -0.01 |  | -0.20 | 0.02 |  | -0.14 | 0.01 |  | -0.07 | -0.02 |  | -0.12 | 0.02 |
| disability | 0.102 | 0.003 |  | 0.04 | 0.06 |  | 0.02 | 0.01 |  | 0.03 | 0.03 |  | -0.01 | 0.04 |  | -0.01 | 0.04 |
| foreign | 0.022 | 0.069 |  | 0.01 | 0.05 |  | -0.04 | 0.01 |  | -0.03 | 0.00 |  | -0.01 | 0.02 |  | -0.05 | 0.01 |
| 4-year degree | 0.020 | 0.041 |  | -0.07 | 0.03 |  | -0.09 | 0.01 |  | -0.12 | 0.03 |  | -0.08 | 0.00 |  | -0.08 | 0.02 |
| 3-year degree | -0.038 | -0.007 |  | 0.10 | -0.02 |  | 0.19 | -0.05 |  | 0.03 | -0.03 |  | 0.15 | -0.05 |  | 0.27 | -0.01 |
| part-time job | - | - |  | - | - |  | 0.35 | 0.04 |  | 0.24 | 0.04 |  | 0.71 | 0.02 |  | 0.55 | 0.03 |
| Andalucía | 0.000 | -0.008 |  | 0.19 | -0.02 |  | 0.07 | 0.00 |  | 0.01 | 0.00 |  | 0.11 | -0.03 |  | 0.08 | -0.01 |
| Aragón | 0.016 | 0.007 |  | -0.06 | -0.01 |  | 0.01 | 0.01 |  | -0.02 | -0.01 |  | -0.04 | -0.01 |  | 0.03 | 0.02 |
| Asturias (Princ. de) | 0.019 | -0.020 |  | 0.06 | 0.04 |  | 0.02 | 0.01 |  | 0.00 | 0.05 |  | 0.01 | 0.02 |  | 0.02 | 0.00 |
| Balears (Illes) | 0.013 | -0.010 |  | -0.03 | 0.01 |  | 0.02 | 0.00 |  | -0.03 | 0.01 |  | -0.05 | 0.00 |  | 0.02 | 0.02 |
| Canarias | 0.086 | 0.011 |  | 0.07 | -0.01 |  | 0.06 | 0.01 |  | 0.04 | 0.02 |  | 0.08 | 0.02 |  | 0.01 | -0.01 |
| Cantabria | -0.024 | -0.015 |  | 0.01 | 0.01 |  | 0.00 | 0.00 |  | -0.02 | 0.02 |  | 0.05 | -0.02 |  | 0.04 | 0.00 |
| Castilla y León | -0.004 | -0.009 |  | 0.05 | -0.01 |  | 0.00 | 0.00 |  | 0.01 | 0.01 |  | 0.06 | 0.00 |  | 0.01 | 0.01 |
| Castilla-La Mancha | -0.021 | 0.003 |  | 0.07 | 0.01 |  | 0.03 | -0.01 |  | 0.03 | -0.01 |  | 0.02 | -0.01 |  | 0.05 | 0.01 |
| Cataluña | -0.027 | 0.023 |  | -0.23 | 0.00 |  | -0.09 | -0.01 |  | -0.08 | -0.03 |  | -0.13 | -0.02 |  | -0.07 | -0.01 |
| Comunitat Valenciana | 0.012 | -0.005 |  | 0.03 | 0.01 |  | 0.03 | -0.01 |  | -0.01 | -0.01 |  | 0.05 | 0.05 |  | 0.01 | -0.01 |
| Extremadura | -0.024 | 0.000 |  | 0.09 | 0.02 |  | 0.04 | 0.00 |  | 0.03 | 0.02 |  | 0.09 | 0.00 |  | 0.05 | -0.04 |
| Galicia | 0.000 | -0.004 |  | 0.07 | -0.01 |  | 0.04 | -0.02 |  | 0.00 | 0.03 |  | 0.05 | 0.01 |  | 0.02 | -0.01 |
| Murcia (Región de) | 0.040 | 0.015 |  | 0.04 | 0.00 |  | 0.02 | 0.00 |  | 0.03 | 0.02 |  | 0.05 | 0.02 |  | 0.01 | 0.01 |
| Navarra(Com. Foral) | 0.023 | 0.020 |  | -0.05 | 0.01 |  | -0.05 | 0.01 |  | -0.07 | 0.01 |  | -0.01 | -0.01 |  | 0.02 | -0.01 |
| País Vasco | -0.014 | -0.017 |  | -0.03 | -0.01 |  | -0.02 | 0.02 |  | -0.01 | 0.01 |  | 0.04 | -0.01 |  | 0.08 | 0.00 |
| Rioja (La) | 0.020 | 0.000 |  | -0.05 | 0.01 |  | 0.03 | -0.02 |  | 0.00 | -0.02 |  | -0.04 | -0.02 |  | 0.02 | -0.01 |
| Distance universities | 0.062 | -0.002 |  | -0.06 | 0.00 |  | 0.06 | 0.00 |  | 0.25 | -0.05 |  | -0.12 | 0.01 |  | -0.11 | 0.02 |
| Early education training | 0.010 | 0.005 |  | 0.03 | 0.00 |  | 0.10 | -0.03 |  | -0.03 | -0.01 |  | 0.19 | 0.02 |  | 0.20 | 0.01 |
| Primary edu. teacher training | -0.016 | 0.003 |  | 0.05 | -0.01 |  | 0.06 | -0.02 |  | 0.03 | -0.01 |  | 0.15 | -0.03 |  | 0.18 | 0.00 |
| Other training of teaching staff | 0.024 | -0.016 |  | -0.01 | -0.01 |  | 0.03 | 0.00 |  | 0.02 | -0.01 |  | 0.07 | -0.03 |  | 0.20 | 0.02 |
| Arts | 0.091 | -0.017 |  | 0.11 | 0.01 |  | 0.12 | 0.02 |  | 0.11 | 0.02 |  | 0.16 | 0.03 |  | 0.01 | 0.01 |
| Audiovisual techn. & media | 0.023 | 0.011 |  | 0.06 | 0.00 |  | 0.08 | 0.01 |  | 0.08 | 0.03 |  | 0.06 | 0.00 |  | 0.01 | -0.03 |
| Humanities | 0.130 | 0.018 |  | 0.13 | -0.01 |  | 0.11 | 0.00 |  | 0.25 | 0.00 |  | 0.05 | 0.01 |  | 0.02 | 0.03 |
| Languages | 0.050 | 0.016 |  | 0.04 | 0.02 |  | -0.04 | -0.03 |  | 0.05 | -0.03 |  | 0.15 | -0.01 |  | 0.10 | -0.02 |
| Psychology | -0.038 | -0.014 |  | 0.03 | -0.01 |  | 0.04 | -0.01 |  | 0.07 | -0.02 |  | 0.10 | 0.00 |  | -0.01 | -0.02 |
| Economics | 0.019 | 0.013 |  | -0.01 | 0.01 |  | 0.05 | -0.01 |  | -0.03 | 0.00 |  | -0.03 | 0.00 |  | -0.05 | 0.00 |
| Other social & behavioral sc. | 0.094 | 0.010 |  | 0.01 | 0.00 |  | 0.03 | 0.02 |  | 0.25 | 0.00 |  | -0.01 | 0.02 |  | -0.04 | 0.03 |
| Journalism | -0.047 | 0.025 |  | 0.06 | 0.02 |  | 0.02 | 0.02 |  | 0.10 | 0.00 |  | 0.07 | 0.01 |  | 0.03 | -0.02 |
| Business admin. & manag. | -0.114 | 0.004 |  | -0.03 | -0.02 |  | 0.16 | -0.01 |  | 0.03 | -0.02 |  | -0.11 | -0.02 |  | -0.14 | -0.01 |
| Other business education | -0.064 | 0.000 |  | -0.01 | -0.01 |  | 0.04 | 0.01 |  | 0.10 | 0.01 |  | 0.00 | 0.01 |  | -0.04 | 0.01 |
| Life sciences | 0.020 | 0.003 |  | 0.12 | -0.01 |  | 0.03 | 0.01 |  | 0.05 | 0.01 |  | 0.04 | 0.01 |  | 0.03 | 0.00 |
| Physical, chemical, geolog. sc. | 0.064 | 0.000 |  | 0.05 | 0.01 |  | -0.02 | 0.03 |  | -0.02 | 0.02 |  | -0.06 | 0.00 |  | 0.01 | 0.00 |
| Mathematics and statistics | -0.010 | -0.004 |  | -0.04 | 0.01 |  | -0.06 | 0.03 |  | -0.02 | 0.05 |  | -0.02 | 0.04 |  | -0.04 | 0.02 |
| Computer Science | -0.101 | -0.003 |  | -0.20 | 0.00 |  | -0.09 | 0.01 |  | -0.22 | 0.00 |  | -0.26 | 0.00 |  | -0.16 | 0.00 |
| Engineering and related prof. | -0.151 | 0.002 |  | -0.12 | 0.02 |  | -0.15 | 0.00 |  | -0.16 | 0.00 |  | -0.26 | 0.00 |  | -0.09 | 0.00 |
| Manufacturing ind. & prod. | -0.128 | 0.013 |  | 0.04 | -0.01 |  | 0.03 | 0.03 |  | 0.05 | 0.02 |  | -0.06 | 0.00 |  | -0.01 | 0.00 |
| Architecture & construction | -0.002 | -0.013 |  | 0.01 | -0.01 |  | -0.05 | 0.01 |  | -0.02 | 0.03 |  | -0.06 | 0.01 |  | -0.11 | -0.01 |
| Agriculture, Livestock and fish. | -0.036 | -0.004 |  | 0.05 | -0.01 |  | 0.02 | -0.01 |  | 0.05 | 0.01 |  | -0.02 | 0.03 |  | 0.01 | -0.01 |
| Veterinary | -0.031 | -0.015 |  | -0.05 | 0.01 |  | -0.12 | 0.03 |  | -0.13 | 0.00 |  | 0.02 | 0.02 |  | -0.06 | 0.00 |
| Medicine | -0.195 | -0.009 |  | -0.24 | 0.00 |  | -0.29 | -0.01 |  | -0.29 | 0.00 |  | -0.27 | 0.00 |  | -0.21 | -0.01 |
| Nursing and care for the sick | 0.030 | 0.002 |  | 0.01 | -0.01 |  | -0.21 | 0.01 |  | -0.25 | 0.00 |  | -0.03 | -0.02 |  | 0.21 | -0.01 |
| Other health sciences | -0.016 | 0.009 |  | -0.12 | -0.01 |  | -0.23 | -0.03 |  | -0.19 | -0.01 |  | 0.02 | 0.00 |  | -0.10 | -0.01 |
| Social work and counseling | -0.006 | 0.016 |  | 0.08 | 0.01 |  | 0.17 | -0.01 |  | 0.17 | -0.03 |  | 0.09 | -0.01 |  | 0.09 | -0.02 |
| Tourism and hospitality | 0.075 | -0.010 |  | 0.04 | 0.01 |  | 0.18 | -0.02 |  | 0.14 | -0.02 |  | 0.05 | -0.03 |  | 0.04 | 0.00 |
| Sports | -0.020 | -0.033 |  | -0.05 | 0.00 |  | 0.06 | 0.00 |  | -0.02 | 0.00 |  | 0.12 | 0.01 |  | 0.10 | 0.02 |
| Transport services | 0.024 | 0.034 |  | 0.02 | 0.04 |  | -0.03 | 0.03 |  | -0.04 | 0.02 |  | -0.03 | 0.00 |  | 0.00 | -0.02 |

Source: University Graduate Job Placement Survey 2014 (INE). Own calculations.
